# Geliboluols A–D: Kaurane-Type Diterpenoids from the Marine-Derived Rare Actinomycete *Actinomadura geliboluensis*

**DOI:** 10.3390/md23020078

**Published:** 2025-02-10

**Authors:** Chang-Su Heo, Jong Soon Kang, Jeong-Wook Yang, Min Ah Lee, Hwa-Sun Lee, Chang Hwan Kim, Hee Jae Shin

**Affiliations:** 1 Marine Natural Products Chemistry Laboratory, Korea Institute of Ocean Science and Technology, 385 Haeyang-ro, Busan 49111, Republic of Korea; science30@kiost.ac.kr (C.-S.H.); minah@kiost.ac.kr (M.A.L.); hwasunlee@kiost.ac.kr (H.-S.L.); 2 Department of Marine Technology and Convergence Engineering, University of Science and Technology (UST), 217 Gajungro, Daejeon 34113, Republic of Korea; 3 Laboratory Animal Resource Center, Korea Research Institute of Bioscience and Biotechnology, 30 Yeongudanjiro, Cheongju 28116, Republic of Korea; kanjon@kribb.re.kr (J.S.K.); z7v8@kribb.re.kr (J.-W.Y.); 4 Department of Chemistry, Pukyong National University, 45 Yongso-ro, Busan 48513, Republic of Korea; 5 Dokdo Research Center, Korea Institute of Ocean Science and Technology, Uljin 36315, Republic of Korea; kimch@kiost.ac.kr

**Keywords:** *Actinomadura geliboluensis*, kaurane, diterpenoids, cytotoxicity, geliboluol

## Abstract

Four new kaurane-type diterpenoids, geliboluols A–D (**1**–**4**), along with one known analog (**5**), were isolated from the culture broth of the marine-derived rare actinomycete *Actinomadura geliboluensis*. The structures of compounds **1**–**4** were determined by spectroscopic analysis (HR-ESIMS, 1D, and 2D NMR), the MPA method, and by comparing their optical rotation values with those in the literature. The new compounds were evaluated for their cytotoxicity against seven blood cancer cell lines by a CellTiter-Glo (CTG) assay and six solid cancer cell lines by a sulforhodamine B (SRB) assay. Among the new compounds, compound **4** exhibited moderate cytotoxic activity against some blood cancer cell lines, with GI_50_ values ranging from 2.59 to 19.64 µM, and against solid cancer cell lines with GI_50_ values ranging from 4.34 to 7.23 µM.

## 1. Introduction

**Figure 1:**
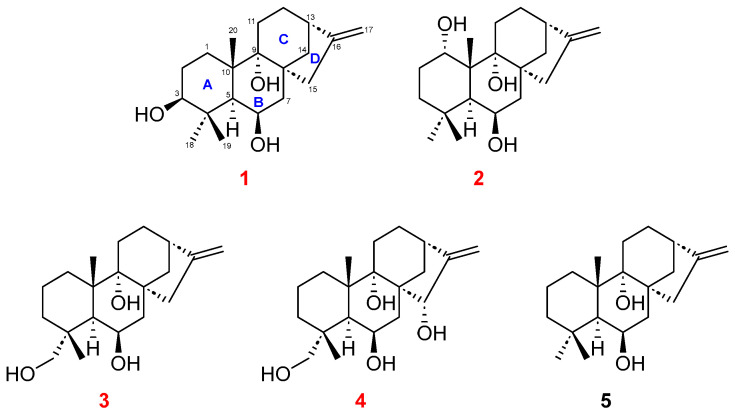
Structures of compounds **1**–**5** isolated from *Actinomadura geliboluensis* 238DD-017.

Diterpenoids, a class of secondary metabolites, are comprised of four isoprene units with twenty carbons. Such diterpenoids are classified as labdanes, pimaranes, kauranes, trachylobane, and so on, depending on the type of cyclization and the number of rings [1,2]. Among diterpenoids, kauranes are tetracyclic diterpenes with four carbon ring moieties [3]. Their structural scaffolds typically consist of a perhydrophenantrene moiety with rings A, B, as well as C (Figure 1), and a cyclopentane moiety with ring D, which is fused at the C-8 and C-13 positions [2]. Depending on the inversion of stereocenters, enantiomers are distinguished into two types, kauranes and *ent*-kauranes. The majority of enantiomers belong to *ent*-kauranes and generally, they have negative values of optical rotation [2]. Most kaurane diterpenoids are discovered in plant sources such as the Lamiaceae family, as well as Asteraceae, Annonaceae, and Euphorbiaceae [3,4]. They are known to be involved in the protective functions of plants that produce them [5,6]. Additionally, their broad biological activities have been reported, including antiparasitic, antifungal, antibacterial, antitumor, anti-inflammatory, and antiviral activities in previous research [4,7,8,9,10,11,12].

Actinobacteria are well-known as an abundant source of secondary metabolites in explorations of novel medicines [13]. They are not only contributors to antibiotic production, such as streptomycin and kanamycin, but are also producers of anti-cancer compounds such as doxorubicin and salinosporamide [14,15]. Among them, *Actinomadura*, a genus of Actinomycetes, has been relatively less-studied than the genus *Streptomyces*, which is common in Actinobacteria. To date, over 70 species of *Actinomadura* have been identified and more than 230 compounds derived from those species have been reported for their unique structures, along with their various bioactivities [16,17,18]. Previous molecular biology studies of the *Actinomadura* genus found that more than 30 or 40 putative biosynthetic gene clusters are located on their genomes [19,20,21], suggesting that most of these are expected to be involved in undiscovered metabolites to date. Therefore, the genus *Actinomadura* has untapped potential in the discovery of novel therapeutic agents [22] and is one of the notable sources of new compounds to explore.

As part of our continuous search for new secondary metabolites from marine microorganisms, an actinobacterial strain was isolated from a sediment sample collected offshore from Dokdo, Republic of Korea. The strain was identified as *Actinomadura geliboluensis* 238DD-017 by 16S rRNA gene sequence analysis. *A. geliboluensis* was first isolated in 2012 in Gelibolu, Canakkale, Turkey [23], and only a few secondary metabolites, such as nomimicins, have been reported from this strain [24]. These facts prompted us to culture this rare strain, and we found the crude extract of the culture broth afforded cytotoxic activity against cancer cell lines. Thus, further study was performed to identify bioactive secondary metabolites produced by the strain. As a result, five kaurane-type diterpenoids, including four new (**1**–**4**) and one known (**5**) derivatives (Figure 1), were isolated from the strain. In this report, the isolation, purification, structure determination, and evaluation of the antimicrobial and cytotoxic activities of the compounds are presented.

## 2. Results

Compound **1** was isolated as a reddish powder. Its molecular formula was determined as C_20_H_32_O_3_, based on a high-resolution electrospray ionization mass spectrometry (HRESIMS) peak at *m*/*z* 343.2239 [M + Na]^+^ (calcd for C_20_H_32_O_3_Na, 343.2244), requiring an index of hydrogen deficiency of five. The ^1^H NMR data (Table 1) showed signals of two exomethylenes at *δ*_H_ 4.73 (s, H-17a) and 4.77 (s, H-17b), two oxymethines at *δ*_H_ 4.35 (dd, *J* = 5.0, 2.6 Hz, H-6) and 3.07 (dd, *J* = 11.7, 4.3 Hz, H-3), fourteen methylenes at *δ*_H_ 1.32–2.86, two methines at *δ*_H_ 2.59 (overlapped, H-13) and 1.34 (overlapped, H-5), and three methyl groups at *δ*_H_ 1.05–1.51. The ^13^C NMR data (Table 1) and heteronuclear single-quantum coherence (HSQC) NMR data revealed the presence of two olefinic carbons at *δ*_C_ 156.6 (C-16) and 103.0 (C-17), one oxygenated quaternary carbon at *δ*_C_ 79.1 (C-9), two oxymethines at *δ*_C_ 79.8 (C-3) and 68.7 (C-6), two methines at *δ*_C_ 49.5 (C-5) and 44.5 (C-13), three quaternary carbons at *δ*_C_ 48.4 (C-8), 44.6 (C-10), and 40.7 (C-4), seven methylenes at *δ*_C_ 28.2–45.5, and three methyls at *δ*_C_ 28.8 (C-18), 21.8 (C-20), and 17.8 (C-19).

**Figure 2:**
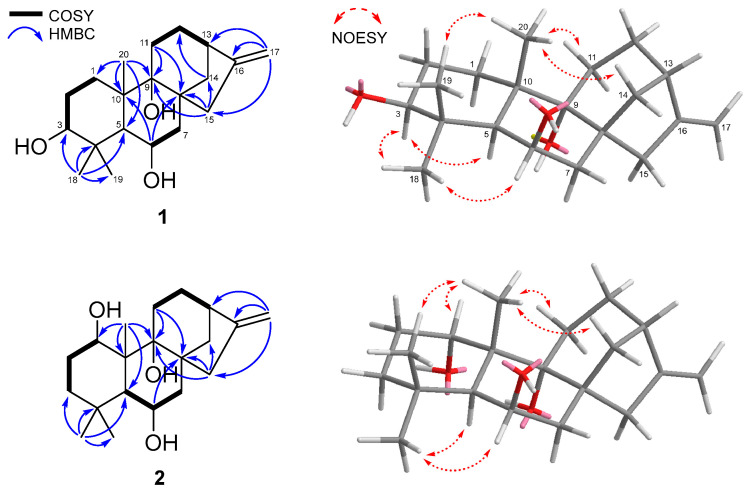
Key COSY, HMBC, and NOESY correlations of compounds **1** and **2**.

The planar structure of **1** was revealed by detailed analyses of ^1^H-^1^H correlation spectroscopy (COSY) and heteronuclear multiple-bond correlation (HMBC) NMR data (Figure 2). The ^1^H-^1^H COSY spectrum exhibited the following three spin systems: H_2_-1/H_2_-2/H-3, H-6/H_2_-7, and H_2_-11/H_2_-12/H-13. The HMBC correlations from H_3_-18 to C-3, C-4, C-5, and C-19 and from H_3_-20 to C-1, C-5, and C-10 confirmed the structure of ring A. The HMBC correlations from H-6 to C-8 and C10 and from H_3_-20 to C-9 established a decalin system of ring B, which was fused with ring A at C-5 and C-10. In addition, the HMBC correlations from H_2_-11 to C-8 and C-9, from H_2_-15 to C-8, C-9, and C-14, from H_2_-14 to C-12, and from H_2_-17 to C-13, C-15, and C-16 elucidated the structure of a tetracyclic diterpenoid with a 6/6/6/5 ring system, including ring C and ring D. Thus, the gross structure of **1** was determined, as shown in Figure 2.

The relative configuration of **1** was elucidated by the analysis of nuclear Overhauser effect spectroscopy (NOESY) data (Figure 2). The NOESY correlations of H-3 (*δ*_H_ 3.07) with H-5 (*δ*_H_ 1.34) and H_3_-18 (*δ*_H_ 1.05) and of H-6 (*δ*_H_ 4.35) with H_3_-18 (*δ*_H_ 1.05) indicated these protons have an α-orientation, while the NOESY correlations of H_3_-19 (*δ*_H_ 1.17) with H_3_-20 (*δ*_H_ 1.51) suggested the *β*-orientation of these protons, and the A/B ring junction was a *trans*-configuration. The NOESY correlations of H_3_-20 with H_b_-11 (*δ*_H_ 1.97) and H_b_-14 (*δ*_H_ 2.60) indicated that they were cofacial and the B/C ring junction was a *cis*-configuration. Therefore, the relative configuration of chiral centers at C-3, C-5, C-6, C-8, C-9, C-10, and C-13 were identified as 3S*, 5S*, 6R*, 8R*, 9R*, 10S*, and 13S*.

**Figure 3:**
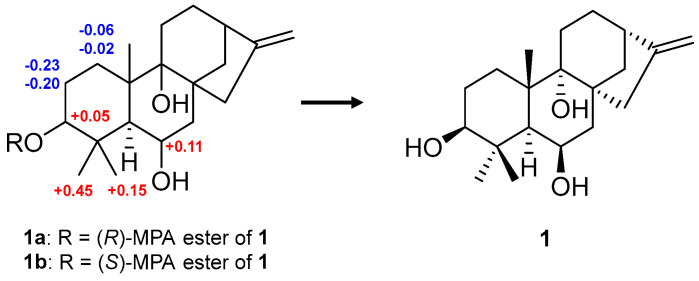
*Δδ_H_* values (in ppm) = *δ_R_*–*δ_S_* obtained for (*R*)- and (*S*)-MPA esters of compound **1**. Data were measured in CD_3_OD.

The absolute configuration of compound **1** was determined based on its optical rotation value and the MPA method. According to the literature, positive optical rotation values are characteristic of kaurane-type diterpenoids [25,26]. On the contrary, negative optical rotation values of kauranes are usually observed in the *ent*-kaurane-type [2,27,28,29,30]. While the majority of kaurane diterpenoids reported to date are on the *ent*-kaurane type [31], unexpectedly, the experimental optical rotation value of **1** was positive (${\left[\alpha \right]}_{D}^{25}$ +37.8, *c* 0.1, MeOH) and was consistent with the kaurane type. Therefore, its absolute configuration was reconfirmed by MPA esterification procedures. By comparing ^1^H NMR data of (*R*)- and (*S*)-MPA esters of **1** (**1a** and **1b**, Figure 3), the absolute configuration of the chiral center at C-3 was identified as 3*S*. Consequently, the absolute configuration of **1** was confirmed as 3*S*, 5*S*, 6*R*, 8*R*, 9*R*, 10*S,* and 13*S*, and **1** was named geliboluol A.

Compound **2** was isolated as a white powder. Its molecular formula of C_20_H_32_O_3_ was confirmed based on the HRESIMS peak at *m*/*z* 319.2273 [M − H]^−^ (calcd for C_20_H_31_O_3_, 319.2279), indicating that it was identical to that of **1**. The ^1^H and ^13^C NMR spectra of **2** were similar to those of **1**, except for chemical shifts in **2** at C-1 (*δ*_H_ 3.94/*δ*_C_ 75.4 for **2** and *δ*_H_ 1.39, 1.88/*δ*_C_ 33.5 for **1**) and C-3 (*δ*_H_ 1.03, 1.78/*δ*_C_ 37.6 for **2** and *δ*_H_ 3.07/*δ*_C_ 79.8 for **1**). Comparing the chemical shifts of **2** with those of **1** suggested that the structure of **2** was similar to that of **1,** with a different position for an hydroxy group. Through detailed analysis of the HSQC, COSY, and HMBC data, the hydroxy group of **2** was assigned at C-1 and the planar structure was determined as depicted in Figure 2. The relative configuration of **2** was confirmed by the interpretation of the NOESY data. The NOESY correlations of **2** were similar to those of **1** and the *β*-orientation of H-1 was established (Figure 2), revealing the relative configuration of **2** as 1S*, 5S*, 6R*, 8R*, 9R*, 10R*, and 13S*. The positive optical rotation value of **2** (${\left[\alpha \right]}_{D}^{25}$ +29.9, *c* 0.1, MeOH) indicated that **2** is also a kaurane-type diterpenoid. Therefore, the absolute configuration of **2** was elucidated as 1*S*, 5*S*, 6*R*, 8*R*, 9*R*, 10*R,* and 13*S* and given the name geliboluol B.

**Figure 4:**
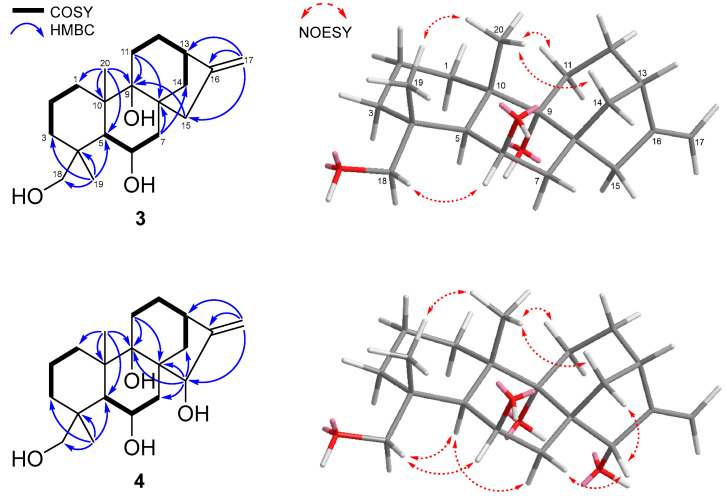
Key COSY, HMBC, and NOESY correlations of compounds **3** and **4**.

Compound **3** was isolated as a white powder. Its molecular formula of C_20_H_32_O_3_ was determined based on the HRESIMS peak at *m*/*z* 343.2229 [M + Na]^+^ (calcd for C_20_H_32_O_3_Na, 343.2227), revealing that it was consistent with those of **1** and **2**. The ^1^H and ^13^C NMR data of **3** resembled those of **1**, except for the absence of a singlet methyl signal at the C-18 position (*δ*_H_ 3.10, 3.53/*δ*_C_ 34.2 for **3** and *δ*_H_ 1.05/*δ*_C_ 28.8 for **1**). Accordingly, it was suggested that the methyl group at C-18 in **1** was changed to an oxygenated methylene in **3**. Further detailed analysis of the HSQC, COSY, and HMBC data confirmed the planar structure of **3** (Figure 4) and the interpretation of NOESY data elucidated the relative structure as 4R*, 5S*, 6R*, 8R*, 9R*, 10S*, and 13S*, as depicted in Figure 4. The optical rotation value of **3** was positive (${\left[\alpha \right]}_{D}^{25}$ +43.5, *c* 0.1, MeOH), and thus the absolute configuration was confirmed as 4*R*, 5*S*, 6*R*, 8*R*, 9*R*, 10*S,* and 13*S*, and **3** was named geliboluol C.

Compound **4** was isolated as a white powder with a molecular formula of C_20_H_32_O, as determined by the HRESIMS peak at *m*/*z* 359.2187 [M + Na]^+^ (calcd for C_20_H_32_O_4_Na, 359.2193), suggesting the presence of one more oxygen atom in **4** compared to **3**. The ^1^H and ^13^C NMR data of **4** were similar to those of **3**, indicating that the structure of **4** was similar to that of **3**. The only difference was an additional oxymethine signal in **4** at the C-15 position (*δ*_H_ 3.90/*δ*_C_ 86.7 for **4**, and *δ*_H_ 1.68, 2.89/*δ*_C_ 45.7 for **3**). The interpretation of 2D NMR data (HSQC, COSY, and HMBC) revealed the planar structure of **4**. The methylene at the C-15 position of **3** was changed to an oxygenated methine in **4**, as shown in Figure 4. The detailed analysis of the NOESY data established the relative structure of **4** (Figure 4). The NOESY correlations of H-15 (*δ*_H_ 3.90) with H_a_-14 (*δ*_H_ 1.23) and H_a_-7 (*δ*_H_ 1.22) indicated that they were cofacial, and therefore, the *β*-orientation of H-15 was confirmed. The absolute configuration of **4** was determined as 4*R*, 5*S*, 6*R*, 8*S*, 9*S*, 10*S*, 13*S,* and 15*S* based on its positive optical rotation value (${\left[\alpha \right]}_{D}^{25}$ +59.9, *c* 0.1, MeOH) and given the name geliboluol D.

### Bioactivities

Compounds **1**–**5** were evaluated for their cytotoxicity against seven blood cancer cell lines, the most common cancer types in Korea, along with a normal cell line: HL-60 (acute myelogenous leukemia, AML), Raji (Burkitt’s lymphoma), WSU-DLCL2 (diffuse large B-cell lymphoma, DLBCL), NALM6 C. G5 (B-cell acute lymphocytic leukemia, B-ALL), K562 (chronic myelogenous leukemia, CML), RPMI-8402 (T-cell acute lymphocytic leukemia, T-ALL), U266 (multiple myeloma), and RPMI-1788 (B lymphocytes, normal cell line). Compounds **4** and **5** exhibited cytotoxic activity against blood cancer cell lines with GI _50_ values ranging from 2.59 to 22.50 µM (Table 2), while other compounds did not show cytotoxicity against standard cell lines (GI _50_: >30 µM).

Compounds **1**–**5** were further evaluated for their cytotoxicity against six solid cancer cell lines and a normal cell line: ACHN (renal), MDA-MB-231 (breast), PC-3 (prostate), NUGC-3 (stomach), NCI-H23 (lung), HCT-15 (colon), and MRC-9 (lung fibroblast, normal cell line). Only compound **4** showed cytotoxicity against the six solid cancer cell lines or the normal cell line with GI_50_ values ranging from 4.34 to 7.23 µM (Table 3), while other compounds did not (GI_50_: >30 µM). When comparing GI_50_ values of **4** with those of the previous studies [10,32], our result displayed a similar tendency. It is noteworthy that **5** has more selective activity against blood cancer cell lines than **4**. Additionally, compounds **1**–**5** were evaluated for their antimicrobial properties by applying the broth microdilution method, described by the Clinical and Laboratory Standards Institute [33], against three Gram-positive bacteria, *Bacillus subtilis* (KCTC 1021), *Micrococcus luteus* (KCTC 1915), and *Staphylococcus aureus* (KCTC 1927), and three Gram-negative bacteria, *Escherichia coli* (KCTC 2441), *Salmonella enterica* serovar Typhimurium (KCTC 2515), and *Klebsiella pneumoniae* (KCTC 2690). However, none of the compounds exhibited significant activity against standard bacteria.

## 3. Materials and Methods

### 3.1. General Experimental Procedures

One-dimensional and two-dimensional NMR data were obtained using a Bruker 600 MHz spectrometer (Bruker BioSpin GmbH, Rheinstetten, Germany). High-resolution ESIMS data were acquired with a Sciex X500R Q-TOF spectrometer (Sciex, Framingham, MA, USA). Low-resolution ESIMS data were obtained using an ISQEM mass spectrometer (Thermo Fisher Scientific, Waltham, MA, USA). UV–VIS spectra were measured with a Thermoscientific GENESYS 180 spectrophotometer (Thermo Fisher Scientific, Waltham, MA, USA). IR spectra were recorded using a JASCO FT/IR-4100 spectrophotometer (JASCO Corporation, Tokyo, Japan). Optical rotation values were measured with a Rudolph analytical Autopol III S2 polarimeter with a sodium D line at 589 nm and a 10 mm path length (Rudolph Research Analytical, Hackettstown, NJ, USA). HPLC experiments were performed using a BLS-Class pump (Teledyne SSI, Inc., State College, PA, USA) on an ODS column (YMC-Pack-ODS-A, 250 × 10 mm i.d, 5 µm, Kyoto, Japan) with a Shodex RI-201H refractive index detector (Shoko Scientific Co., Ltd., Yokohama, Japan).

### 3.2. Isolation and Identification of the Strain 238DD-017

The strain 238DD-017 was isolated from a sediment sample collected off-shore of Dokdo, Republic of Korea, during expeditions in August 2023. Aboard the ship, the sediment was collected by a piston corer, and the sample was put into sterile 50 mL conical tubes and stored at 5 °C for transport to the laboratory. Then, to eliminate unwanted microorganisms, heating pretreatment was performed. Each 1 g of sediment sample was placed on a sterile plate and kept in an oven at 60 °C for 30 min. After the heating pretreatment, 0.1 g of sediment was serially diluted to 10^−1^, 10^−2^, and 10^−3^ by sterile seawater, and each diluent (50 µL) was spread on humic acid vitamin (HV) agar, actinomycetes isolation agar (AIA), and on Bennett’s (BN) agar media. The agar plates were incubated in a BOD (Bio-Oxygen Demand) incubator at 28 °C for 7~28 days until colonies appeared. After incubation, the selected colonies were transferred onto new BN agar plates and purification was performed several times until single pure colonies were visible. The strain 238DD-017 was isolated from the HV agar and incubated for 7 days. The strain was identified as *Actinomadura geliboluensis* based on 16S rRNA gene sequence analysis by Macrogen Inc. (Seoul, Republic of Korea). The sequence of 238DD-017 was registered in GenBank (GenBank accession number PQ312698).

### 3.3. Fermentation of the Strain 238DD-017

The seed and mass cultures of the strain 238DD-017 were carried out using a BN medium (glucose 10 g, tryptone 2 g, yeast extract 1 g, beef extract 1 g, glycerol 5 g, and sea salt 32 g/L). A single colony of the strain from an agar plate was aseptically inoculated into a 100 mL conical flask filled with 50 mL of BN broth medium and incubated at 28 °C for 14 days on a rotary shaker at 140 rpm. The broth culture (50 mL) was aseptically transferred to a 2 L flask containing 1 L of BN broth, and the strain was incubated at 28 °C for 14 days on a rotary shaker at 120 rpm. The seed culture broth was inoculated into a 100 L fermenter filled with 70 L of BN broth and incubated at 28 °C for 14 days.

### 3.4. Extraction and Isolation of Metabolites

The mass culture broth (70 L) was harvested and separated into supernatant and mycelium by centrifugation at 60,000 rpm. The supernatant was extracted twice with an equal volume of ethyl acetate (EtOAc, 70 L × 2) and the EtOAc layer was evaporated to yield a crude extract (8.8 g). The crude extract was loaded into ODS column chromatography followed by a stepwise gradient elution with methanol (MeOH) in H_2_O (1:4, 2:3, 3:2, 4:1, and 10:0, *v*/*v*). The fraction eluted with MeOH/H_2_O (4:1, 92.3 mg) was purified by a semi-preparative reversed-phase HPLC (YMC-Pack-ODS-A, 250 × 10 mm i.d, 5 µm; flow rate: 2.0 mL/min; detector: RI) using isocratic elutions of 65% MeOH in H_2_O to yield compound **1** (3.8 mg, *t*_R_ = 28 min) and 70% MeOH in H_2_O to yield compounds **2** (1.0 mg, *t*_R_ = 42 min), **3** (1.5 mg, *t*_R_ = 40 min), and **4** (1.0 mg, *t*_R_ = 34 min). The fraction eluted with MeOH/H_2_O (10:0, 438.1 mg) was purified by a semi-preparative reversed-phase HPLC (YMC-Pack-ODS-A, 250 × 10 mm i.d, 5 µm; flow rate: 2.0 mL/min; detector: RI) using an isocratic elution of 85% MeOH in H_2_O to yield compound **5** (1.9 mg, *t*_R_ = 19 min).

Geliboluol A (**1**): a reddish powder; ${\left[\alpha \right]}_{D}^{25}$ +37.8 (*c* 0.1, MeOH); UV (MeOH) *λ*_max_ (log *ε*) 210 (3.08) nm; IR *ν*_max_ 3491, 2948, 1056 cm^−1^; HRESIMS *m*/*z* 343.2239, [M + Na]^+^, calcd for C_20_H_32_O_3_Na 343.2244. For ^1^H NMR (CD_3_OD, 600 MHz), see Table 1. For ^13^C NMR (CD_3_OD, 150 MHz), see Table 1.

Geliboluol B (**2**): a white powder; ${\left[\alpha \right]}_{D}^{25}$ +29.9 (*c* 0.1, MeOH); UV (MeOH) *λ*_max_ (log *ε*) 209 (3.00) nm; IR *ν*_max_ 3498, 2924, 1015 cm^−1^; HRESIMS *m*/*z* 319.2273, [M-H]^-^, calcd for C_20_H_31_O_3_ 319.2279. For ^1^H NMR (CD_3_OD, 600 MHz), see Table 1. For ^13^C NMR (CD_3_OD, 150 MHz), see Table 1.

Geliboluol C (**3**): a white solid; ${\left[\alpha \right]}_{D}^{25}$ +43.5 (*c* 0.1, MeOH); UV (MeOH) *λ*_max_ (log *ε*) 210 (3.08) nm; IR *ν*_max_ 3396, 1043 cm^−1^; HRESIMS *m*/*z* 343.2229, [M + Na]^+^, calcd for C_20_H_32_O_3_Na 343.2227. For ^1^H NMR (CD_3_OD, 600 MHz), see Table 1. For ^13^C NMR (CD_3_OD, 150 MHz), see Table 1.

Geliboluol D (**4**): a white powder; ${\left[\alpha \right]}_{D}^{25}$ +59.9 (*c* 0.1, MeOH); UV (MeOH) *λ*_max_ (log *ε*) 209 (2.88) nm; IR *ν*_max_ 2898, 1049 cm^−1^; HRESIMS *m*/*z* 359.2187, [M + Na]^+^, calcd for C_20_H_32_O_4_Na 359.2193. For ^1^H NMR (CD_3_OD, 600 MHz), see Table 1. For ^13^C NMR (CD_3_OD, 150 MHz), see Table 1.

Compound **5**: a white powder; ${\left[\alpha \right]}_{D}^{25}$ +63.2 (*c* 0.1, MeOH); UV (MeOH) *λ*_max_ (log *ε*) 208 (3.04) nm; IR *ν*_max_ 3610, 2911 cm^−1^; LRESIMS *m*/*z* 287.30, [M − H_2_O + H]^+^, see Figure S36. For ^1^H NMR (CD_3_OD, 600 MHz), see Figure S37. For ^13^C NMR (CD_3_OD, 150 MHz), see Figure S38.

### 3.5. Preparation of MPA Esters of Compound **1**

(*R*)-α-methoxyphenylacetic acid (MPA) (2.5 mg), *N*,*N*′-dicyclohexylcarbodiimide (DCC) (1.5 mg), and 4-dimethylaminopyridine (DMAP) (0.8 mg) were added to a solution of compound **1** (0.3 mg) in methylene chloride (1.0 mL) to obtain the (*R*)-MPA ester of compound **1** (**1a**). The mixture was stirred at room temperature for 72 h. The reaction mixture was then dried under a N_2_ gas steam at 38 °C and was extracted with EtOAc. The extract was purified by reversed-phase HPLC to obtain **1a**. (*S*)-MPA ester of **1** (**1b**) was also acquired by the same procedure with (*S*)-α-methoxyphenylacetic acid (MPA). NMR data were measured in CD_3_OD.

(*R*)-MPA ester of **1** (**1a**): *δ*_H_ 7.45-7.35 (MPA-Ar), 4.85 (MPA-H), 4.77, 4.73 (H-17), 4.42 (H-3), 4.31 (H-6), 3.40 (MPA-OMe), 2.84, 1.69 (H-15), 2.58 (H-13), 2.57, 1.36 (H-14), 2.03, 1.32 (H-11), 1.91, 1.32 (H-7), 1.89, 1.34 (H-1), 1.78, 1.55 (H-12), 1.61, 1.41 (H-2), 1.48 (H-20), 1.43 (H-5), 1.24 (H-19), and 0.91 (H-18).

(*S*)-MPA ester of **1** (**1b**): *δ*_H_ 7.44-7.33 (MPA-Ar), 4.83 (MPA-H), 4.77, 4.72 (H-17), 4.37 (H-3), 4.20 (H-6), 3.38 (MPA-OMe), 2.83, 1.67 (H-15), 2.57 (H-13), 2.55, 1.34 (H-14), 1.94, 1.32 (H-11), 1.97, 1.27 (H-7), 1.91, 1.40 (H-1), 1.78, 1.54 (H-12), 1.81, 1.64 (H-2), 1.51 (H-20), 1.35 (H-5), 1.09 (H-19), and 0.46 (H-18).

### 3.6. CellTiter-Glo (CTG) and Sulforhodamine B (SRB) Assay for Cytotoxicity Testing

The cytotoxicity test was conducted with CTG luminescent cell viability and SRB assay according to the published procedures [34,35]. Briefly, blood cancer cell lines and a normal cell line were obtained from the American Type Culture Collection (Manassas, VA, USA) (HL-60: acute myelogenous leukemia, Raji: Burkitt’s lymphoma, NALM6 C. G5: B-cell acute lymphocytic leukemia, K562: chronic myelogenous leukemia, U266: multiple myeloma, and RPMI-1788: B lymphocytes) and the DSMZ-German Collection of Microorganisms and Cell Cultures (WSU-DLCL2: diffuse large B-cell lymphoma and RPMI-8402: T-cell acute lymphocytic leukemia). The cell lines were incubated in RPMI 1640 supplemented with 10% fetal bovine serum, penicillin (100 IU/mL), and streptomycin (100 µg/mL) at 37 °C under a humidified atmosphere of 5% CO_2_. The cells with a passage number between 8 and 12 were used. The cell lines were prepared in an opaque-walled 96-well plate (8 × 10^3^ cells/well), and the compounds (**1**–**5** and doxorubicin as a positive control) with 0.1% DMSO were added to each well and incubated for 48 h. The cell cultures were then treated with 100 µL of CellTiter-Glo Reagent (Promega, Madison, WI, USA) and kept for 10 min to acquire a luminescence signal. Luminescence measurements were performed with the GloMax-Multi Detection System (Promega, Madison, WI, USA). The solid cancer cell lines and the normal cell line were obtained from the American Type Culture Collection (Manassas, VA, USA) (ACHN: renal, MDA-MB-231: breast, PC-3: prostate, NCI-H23: lung, HCT-15: colon, and MRC-9: lung fibroblast) and the Japanese Cancer Research Resources Bank (JCRB) (NUGC-3: stomach). The cell lines were incubated in RPMI 1640 supplemented with 10% fetal bovine serum, penicillin (100 IU/mL), and streptomycin (100 µg/mL) at 37 °C under a humidified atmosphere of 5% CO_2_. The cells with a passage number between 8 and 12 were used. The cells were then prepared in a 96-well plate (8 × 10^3^ cells/well), and the compounds (**1**–**5** and adriamycin as a positive control) with 0.1% DMSO were added to each well. After incubation for 48 h, the cell cultures were fixed using 50% trichloroacetic acid (50 µg/mL) and were dyed with 0.4% SRB in 1% acetic acid. Unbound dye was washed using 1% acetic acid and protein-bound dye was collected with 10 mM Tris base (pH 10.5) to measure optical density. Absorbance was measured at 540 nm with a VersaMax microplate reader (Molecular Devices, Sunnyvale, CA, USA) and GI_50_ values were calculated by GraphPad Prism 4.0 (GraphPad, San Diego, CA, USA).

## 4. Conclusions

In conclusion, five kaurane-type diterpenoids were isolated from the rare actinomycete *Actinomadura geliboluensis* 238DD-017, including four new compounds (**1**–**4**), namely, geliboluols A–D. The structures of the new compounds **1**–**4** were determined by a detailed analysis of the HR-ESIMS and NMR data, and as a result, compounds **1**–**4** were confirmed as kaurane-type diterpenoids, which are uncommon in nature. Compounds **1**–**5** were evaluated for their cytotoxicity. Compounds **4** and **5** showed cytotoxicity against seven blood cancer cell lines with GI_50_ values ranging from 2.59 to 22.50 µM, while against the six solid cancer cell lines, only **4** displayed cytotoxicity, with GI_50_ values ranging from 4.34 to 7.23 µM. These results expand the field of kaurane-type diterpenoids research of the genus *Actinomadura*.

## Figures and Tables

**Table 1:** ^1^H (600 MHz) and ^13^C NMR (150 MHz) data for compounds **1**–**4** in CD_3_OD.

No	1		2		3		4	
	*δ*_H_, Mult (*J* in Hz)	*δ*_C_, Type	*δ*_H_, Mult (*J* in Hz)	*δ*_C_, Type	*δ*_H_, Mult (*J* in Hz)	*δ*_C_, Type	*δ*_H_, Mult (*J* in Hz)	*δ*_C_, Type
1a	1.39, ol	33.5, CH_2_	3.94, t (2.5)	75.4, CH	1.38, ol	35.0, CH_2_	1.28, ol	33.4, CH_2_
1b	1.88, td (13.4, 3.9)				1.72 dd (12.6, 3.0)		1.68, ol	
2a	1.59, m	28.2, CH_2_	1.41, m	28.2, CH_2_	1.50, m	19.3, CH_2_	1.48, m	19.2, CH_2_
2b	1.75, m		2.17, m		1.80, m		1.79, m	
3a	3.07, dd (11.7, 4.3)	79.8, CH	1.03, ddd (13.0, 3.4, 3.3)	37.6, CH_2_	1.14, ol	38.2, CH_2_	1.11, dd (12.3, 3.8)	38.1, CH_2_
3b			1.78, ol		1.46, dd (13.1, 4.0)		1.46, ol	
4		40.7, C		35.1, C		39.7, C		39.6, C
5	1.34, ol	49.5, CH	1.89, d (2.3)	45.8, CH	1.78, ol	44.0, CH	2.02, d (1.9)	43.8, CH
6	4.35, dd (5.0, 2.6)	68.7, CH	4.40, dd (5.6, 3.0)	68.8, CH	4.24, dd (5.0, 2.6)	68.5, CH	4.35, dd (4.7, 2.5)	68.7, CH
7a	1.33, ol	45.3, CH_2_	1.29, ol	45.0, CH_2_	1.29, ol	45.0, CH_2_	1.22, ol	43.6, CH_2_
7b	2.04, dd (14.2, 3.2)		2.17, dd (14.1, 3.2)		2.08, dd (14.2, 3.2)		2.50, dd (13.9, 3.1)	
8		48.4, C		47.9, C		48.5, C		47.4, C
9		79.1, C		81.5, C		79.2, C		82.0, C
10		44.6, C		46.5, C		44.6, C		45.5, C
11a	1.32, ol	29.7, CH_2_	1.57, ol	30.9, CH_2_	1.32, ol	29.6, CH_2_	1.56, m	31.1, CH_2_
11b	1.97, dd (14.5, 5.6)		2.12, m		1.98, dd (14.8, 5.5)		2.10, dd (14.8, 5.5)	
12a	1.55, m	35.7, CH_2_	1.59, m	35.8, CH_2_	1.55, m	35.7, CH_2_	1.65, m	36.8, CH_2_
12b	1.79, m		1.80, m		1.79, m		1.83, m	
13	2.59, ol	44.5, CH	2.59, ol	44.6, CH	2.58, ol	44.8, CH	2.61, ol	41.0, CH
14a	1.38, ol	43.3, CH_2_	1.35, dd (12.7, 4.8)	42.8, CH_2_	1.37, ol	43.4, CH_2_	1.23, ol	39.1, CH_2_
14b	2.60, ol		2.59, ol		2.62, dd (12.6, 2.8)		2.60, ol	
15a	1.70, d (17.2)	45.5, CH_2_	1.64, d (17.3)	44.9, CH_2_	1.68, d (16.7)	45.7, CH_2_	3.90, t (2.62)	86.7, CH
15b	2.86, d (17.2)		2.84, d (17.3)		2.89, d (16.7)			
16		156.6, C		156.4, C		156.8, C		157.6, C
17a	4.73, s	103.0, CH_2_	4.73, s	103.0, CH_2_	4.72, s	102.9, CH_2_	4.97, s	104.8, CH_2_
17b	4.77, s		4.78, s		4.78, s		5.07, s	
18a	1.05, s	28.8, CH_3_	1.01, s	34.2, CH_3_	3.10, d (11.0)	72.6, CH_2_	3.10, d (11.1)	72.5, CH_2_
18b					3.53, d (11.0)		3.54, d (11.1)	
19	1.17, s	17.8, CH_3_	1.26, s	24.9, CH_3_	1.14, s	20.4, CH_3_	1.13, s	20.4, CH_3_
20	1.51, s	21.8, CH_3_	1.52, s	22.4, CH_3_	1.55, s	22.1, CH_3_	1.53, s	21.7, CH_3_

In this table, “ol” means overlapped.

**Table 2:** Growth inhibition 50% (GI_50_, µM) values of compounds **1**–**5** against blood cancer cell lines.

	Cell Lines	HL-60	Raji	WSU-DLCL2	NALM6 C. G5	K562	RPMI-8402	U266	RPMI-1788
Compounds									
**1**		>30	>30	>30	>30	>30	>30	>30	>30
**2**		>30	>30	>30	>30	>30	>30	>30	>30
**3**		>30	>30	>30	>30	>30	>30	>30	>30
**4**		11.21	2.73	3.48	2.59	19.64	8.51	>30	4.52
**5**		16.42	4.58	5.93	7.34	11.90	11.09	22.50	9.56
Doxorubicin		0.029	0.014	0.008	0.007	0.094	0.035	0.112	0.012

Doxorubicin is used as a positive control.

**Table 3:** Growth inhibition 50% (GI_50_, µM) values of compounds **1**–**5** against solid cancer cell lines.

	Cell Lines	ACHN	MDA-MB-231	PC-3	NUGC-3	NCI-H23	HCT-15	MRC-9
Compounds								
**1**		>30	>30	>30	>30	>30	>30	>30
**2**		>30	>30	>30	>30	>30	>30	>30
**3**		>30	>30	>30	>30	>30	>30	>30
**4**		4.58	5.73	6.50	4.34	5.13	5.73	7.23
**5**		>30	>30	>30	>30	>30	>30	>30
Adriamycin		0.222	0.210	0.106	0.245	0.221	0.305	0.314

Adriamycin is used as a positive control.

## Data Availability

The data presented in this article are available in the Supplementary Materials.

## Supplementary Materials

The following supporting information can be downloaded at: www.mdpi.com/article/10.3390/md23020078/s1. Figures S1–S35: ^1^H, ^13^C, and 2D NMR spectra (COSY, HSQC, HMBC, and NOESY), and HRESIMS data of compounds **1**–**4**. Figures S36–S38: ^1^H, ^13^C NMR, and LR-MS data of compound **5.** Table S1: Cytotoxicity test results of compounds **1**–**5**.
