# Supplementary material for: Geliboluols A–D: Kaurane-Type Diterpenoids from the Marine-Derived Rare Actinomycete Actinomadura geliboluensis
Source: Mar Drugs. 2025 Feb 10;23(2):78. doi: 10.3390/md23020078 (PMC11857663; doi:10.3390/md23020078)
Supplement: Supplementary file 1 [file marinedrugs-23-00078-s001.zip › marinedrugs-3445926-supplementary.pdf]

# Geliboluols A–D: Kaurane-Type Diterpenoids from the Marine-Derived Rare Actinomycete *Actinomadura geliboluensis*


**Chang-Su Heo [1,2], Jong Soon Kang [3], Jeong-Wook Yang [3], Min Ah Lee [1,4], Hwa-Sun Lee [1,4], Chang Hwan Kim [5] and Hee Jae Shin [1,2,*]**

[1] Marine Natural Products Chemistry Laboratory, Korea Institute of Ocean Science and Technology, 385 Haeyang-ro, Busan 49111, Republic of Korea; science30@kiost.ac.kr (C.-S.H.); minah@kiost.ac.kr (M.A.L.); hwasunlee@kiost.ac.kr (H.-S.L.)
[2] Department of Marine Technology and Convergence Engineering, University of Science and Technology (UST), 217 Gajungro, Daejeon 34113, Republic of Korea
[3] Laboratory Animal Resource Center, Korea Research Institute of Bioscience and Biotechnology, 30 Yeongudanjiro, Cheongju 28116, Republic of Korea; kanjon@kribb.re.kr (J.S.K.); z7v8@kribb.re.kr (J.-W.Y.)
[4] Department of Chemistry, Pukyong National University, 45 Yongso-ro, Busan 48513, Republic of Korea
[5] Dokdo Research Center, Korea Institute of Ocean Science and Technology, Uljin 36315, Republic of Korea; kimch@kiost.ac.kr
* Correspondence: shinhj@kiost.ac.kr; Tel.: +82-51-664-3341; Fax: +82-51-664-3340

# Contents

**Figure S1**. Structures of **1-5** isolated from *Actinomadura geliboluensis* 238DD-017. 4

**Figure S2**. HRESIMS data **1**. 5

**Figure S3**. $^{1}$H NMR spectrum of **1** ($CD_3OD$). 6

**Figure S4**. $^{13}$C NMR spectrum of **1** ($CD_3OD$). 7

**Figure S5**. HSQC spectrum of **1** ($CD_3OD$). 8

**Figure S6**. $^{1}$H-$^{1}$H COSY spectrum of **1** ($CD_3OD$). 9

**Figure S7**. HMBC spectrum of **1** ($CD_3OD$). 10

**Figure S8**. NOESY spectrum of **1** ($CD_3OD$). 11

**Figure S9**. $^{1}$H NMR spectrum of (*R*)-MPA ester of **1** (**1a**) ($CD_3OD$). 12

**Figure S10**. HSQC spectrum of (*R*)-MPA ester of **1** (**1a**) ($CD_3OD$). 13

**Figure S11**. COSY spectrum of (*R*)-MPA ester of **1** (**1a**) ($CD_3OD$). 14

**Figure S12**. $^{1}$H NMR spectrum of (*S*)-MPA ester of **1** (**1b**) ($CD_3OD$). 15

**Figure S13**. HSQC spectrum of (*S*)-MPA ester of **1** (**1b**) ($CD_3OD$). 16

**Figure S14**. COSY spectrum of (*S*)-MPA ester of **1** (**1b**) ($CD_3OD$). 17

**Figure S15**. HRESIMS data of **2**. 18

**Figure S16**. $^{1}$H NMR spectrum of **2** ($CD_3OD$). 19

**Figure S17**. $^{13}$C NMR spectrum of **2** ($CD_3OD$). 20

**Figure S18**. HSQC NMR spectrum of **2** ($CD_3OD$). 21

**Figure S19**. $^{1}$H-$^{1}$H COSY NMR spectrum of **2** ($CD_3OD$). 22

**Figure S20**. HMBC NMR spectrum of **2** ($CD_3OD$). 23

**Figure S21**. NOESY NMR spectrum of **2** ($CD_3OD$). 24

**Figure S22**. HRESIMS data of **3**. 25

**Figure S23**. $^{1}$H NMR spectrum of **3** ($CD_3OD$). 26

**Figure S24**. $^{13}$C NMR spectrum of **3** ($CD_3OD$). 27

**Figure S25**. HSQC NMR spectrum of **3** ($CD_3OD$). 28

**Figure S26**. $^{1}$H-$^{1}$H COSY NMR spectrum of **3** ($CD_3OD$). 29

**Figure S27**. HMBC NMR spectrum of **3** ($CD_3OD$). 30

**Figure S28**. NOESY NMR spectrum of **3** ($CD_3OD$). 31

**Figure S29**. HRESIMS data of **4**. 32

**Figure S30**. $^{1}$H NMR spectrum of **4** ($CD_3OD$). 33

**Figure S31**. $^{13}$C NMR spectrum of **4** ($CD_3OD$). 34

**Figure S32**. HSQC NMR spectrum of **4** ($CD_3OD$). 35

**Figure S33**. $^1H$-$^1H$ COSY NMR spectrum of **4** ($CD_3OD$). ........ 36
**Figure S34**. HMBC NMR spectrum of **4** ($CD_3OD$). ........ 37
**Figure S35**. NOESY NMR spectrum of **4** ($CD_3OD$). ........ 38
**Figure S36**. LRESIMS data of **5**. ........ 39
**Figure S37**. $^1H$ NMR spectrum of **5** ($CD_3OD$). ........ 40
**Figure S38**. $^{13}C$ NMR spectrum of **5** ($CD_3OD$). ........ 41
**Table S1**. Results of the cytotoxicity test of **1-5**. ........ 43

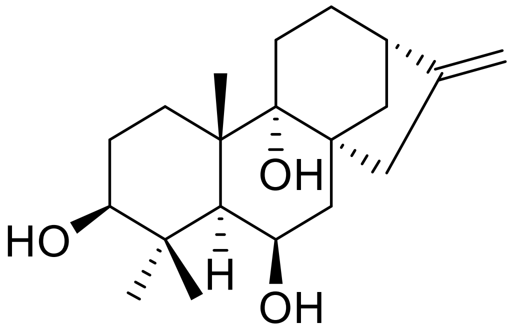

**1**

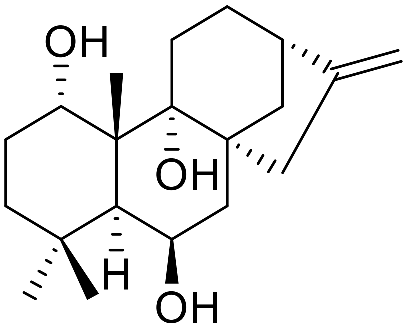

**2**

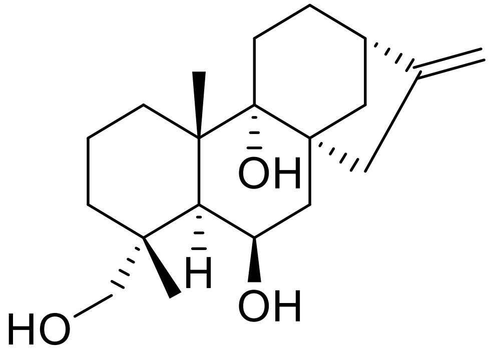

**3**

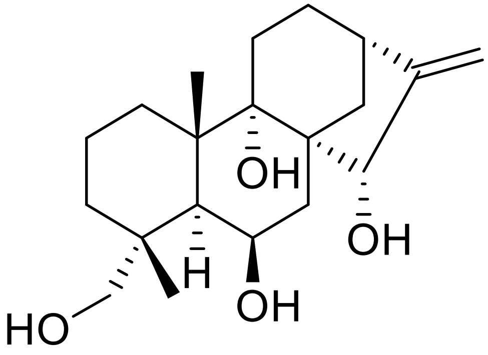

**4**

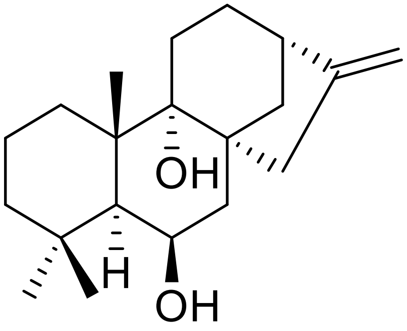

**5**

**Figure S1**. Structures of **1-5** isolated from *Actinomadura geliboluensis* 238DD-017.

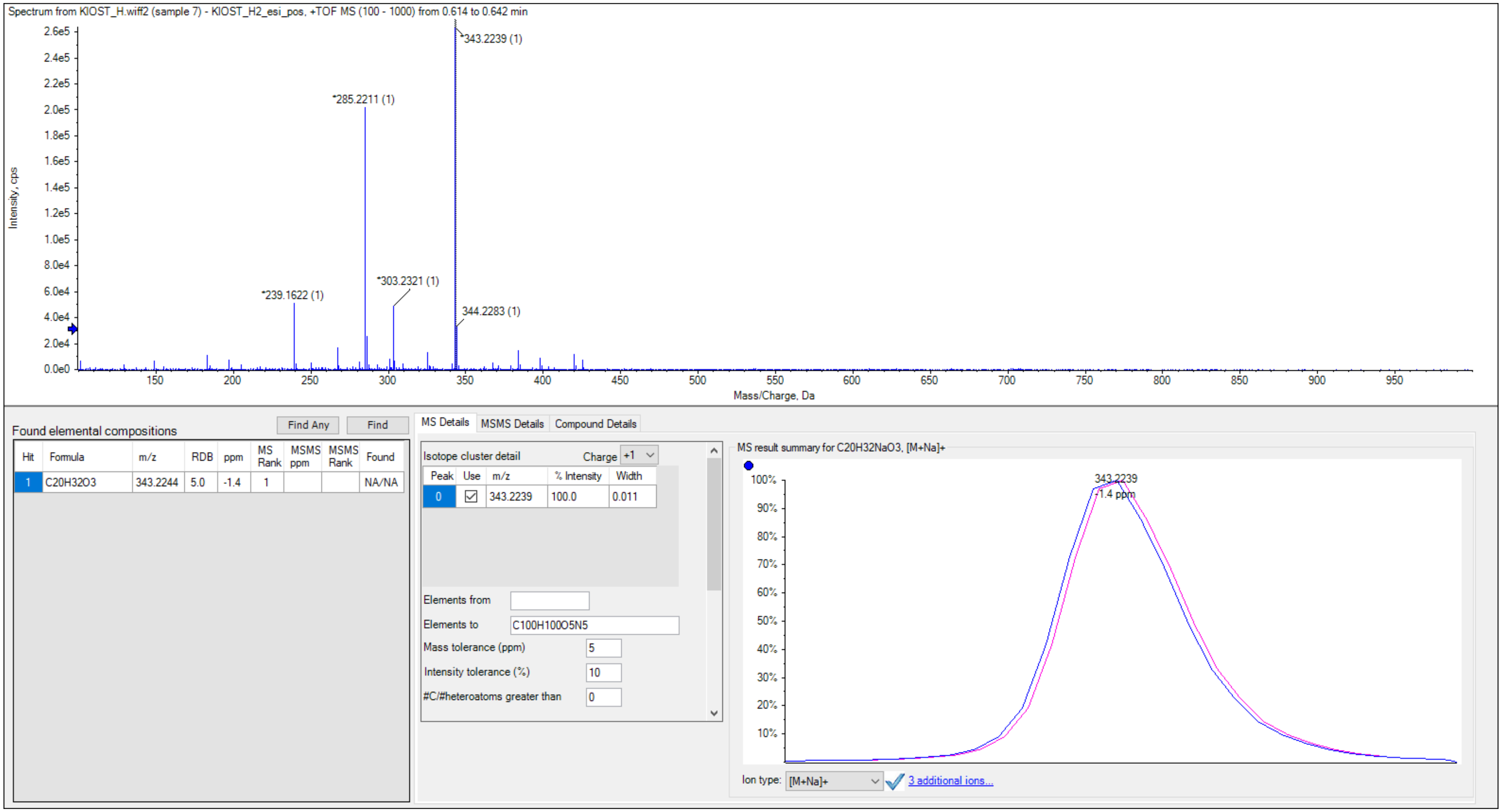


**Figure S2**. HRESIMS data **1**.

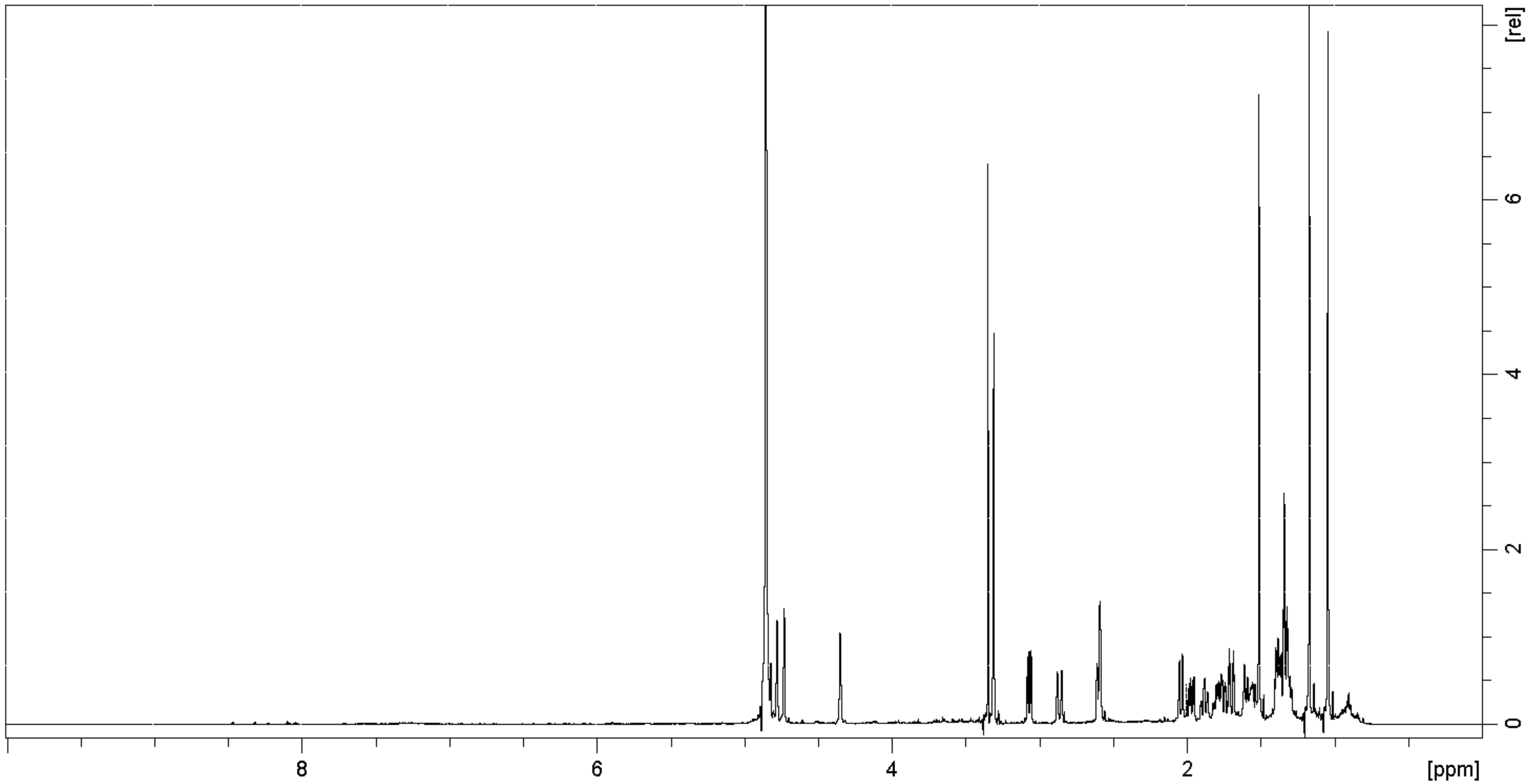


**Figure S3**. $^{1}H$ NMR spectrum of **1** ($CD_3OD$).

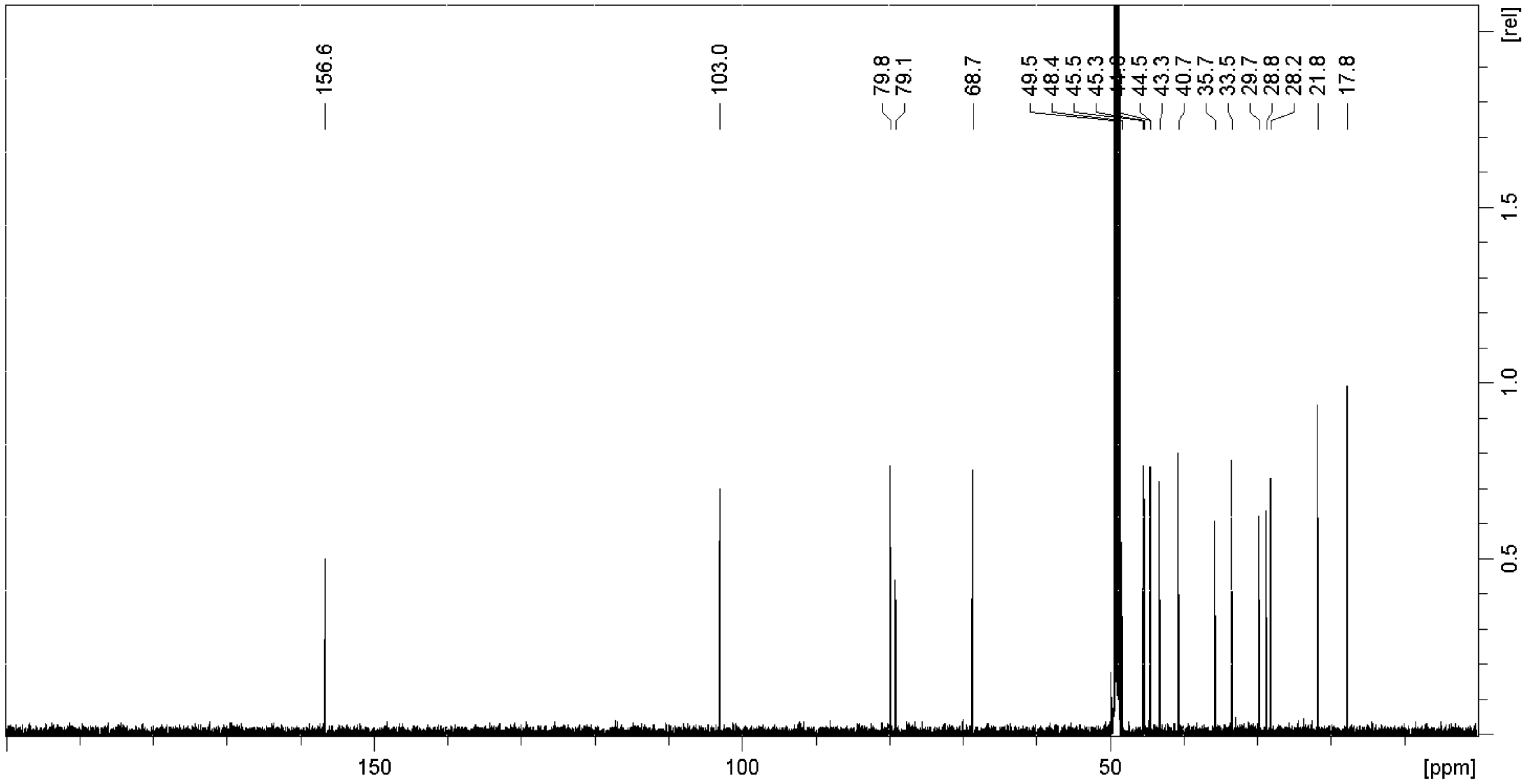


**Figure S4**. $^{13}$C NMR spectrum of **1** ($CD_3OD$).

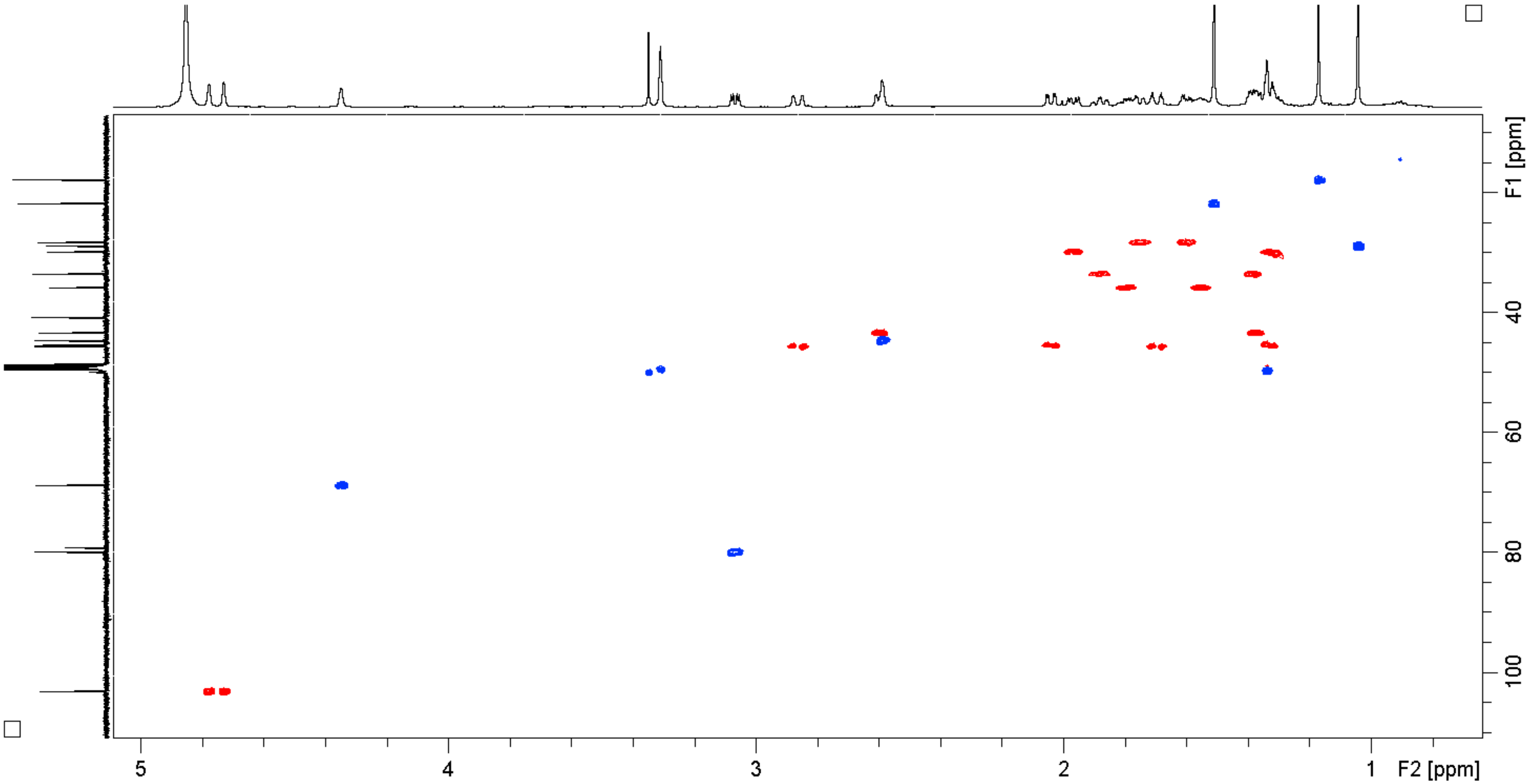


**Figure S5**. HSQC spectrum of **1** ($CD_3OD$).

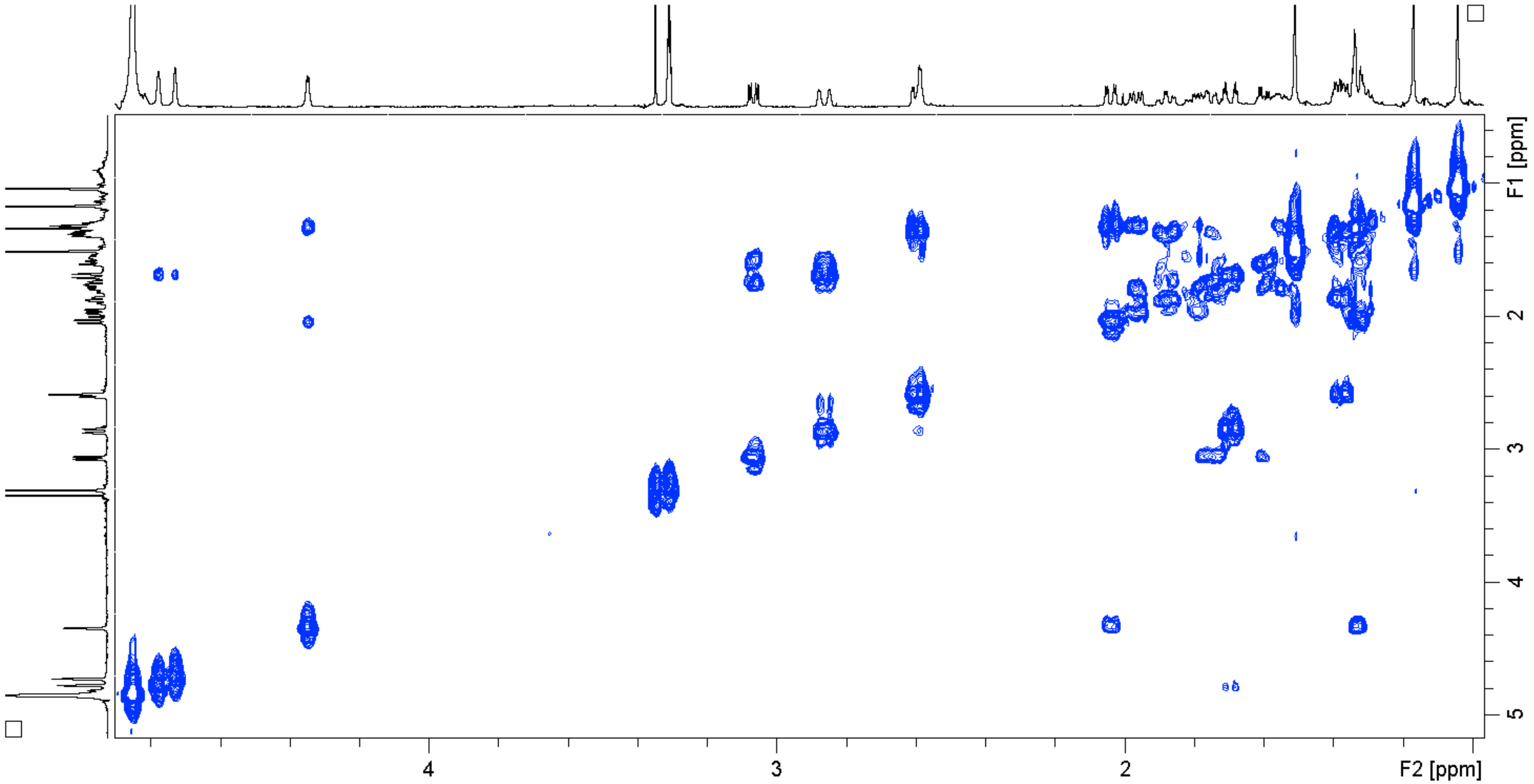


**Figure S6**. $^1H$-$^1H$ COSY spectrum of **1** ($CD_3OD$).

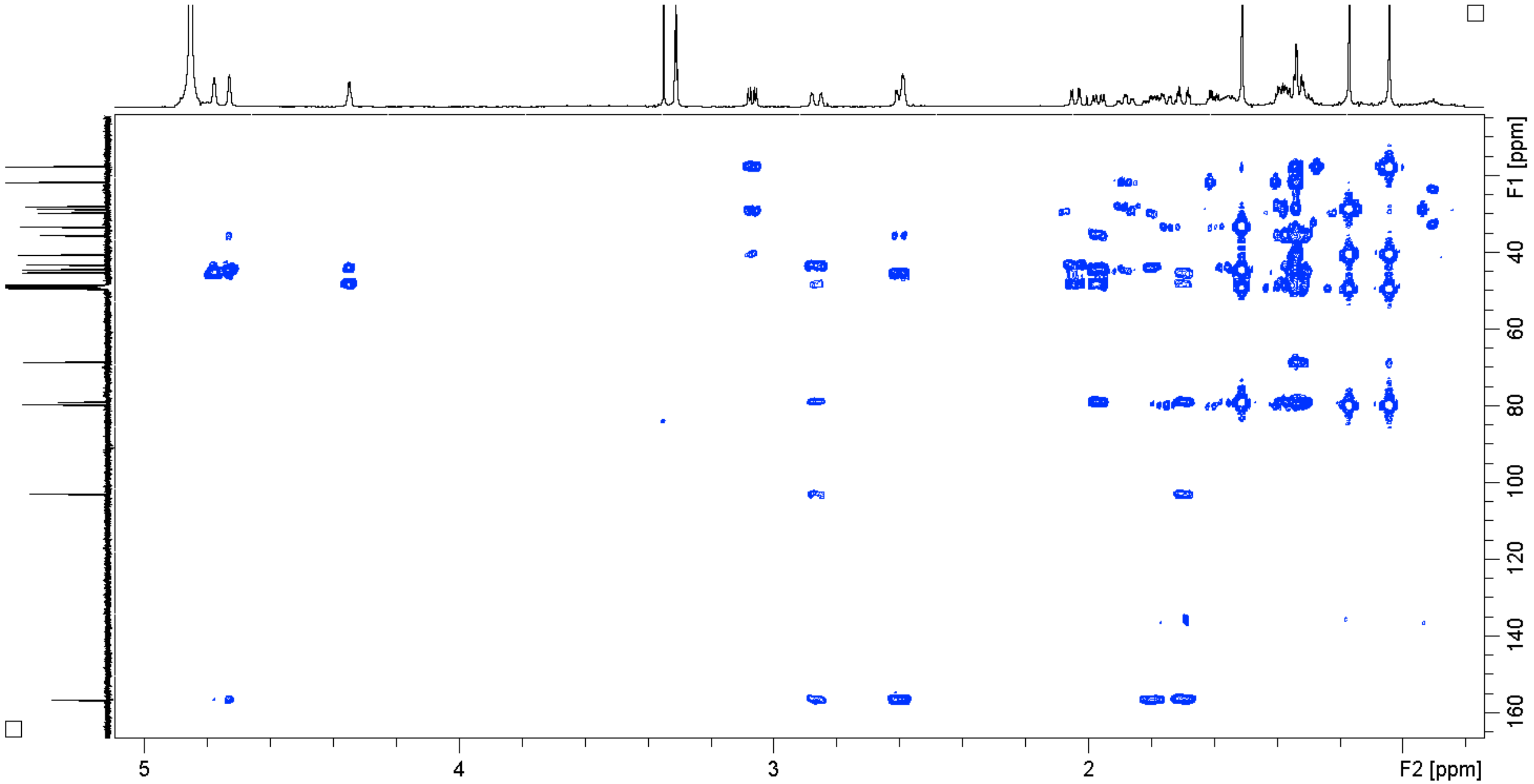


**Figure S7**. HMBC spectrum of **1** ($CD_3OD$).

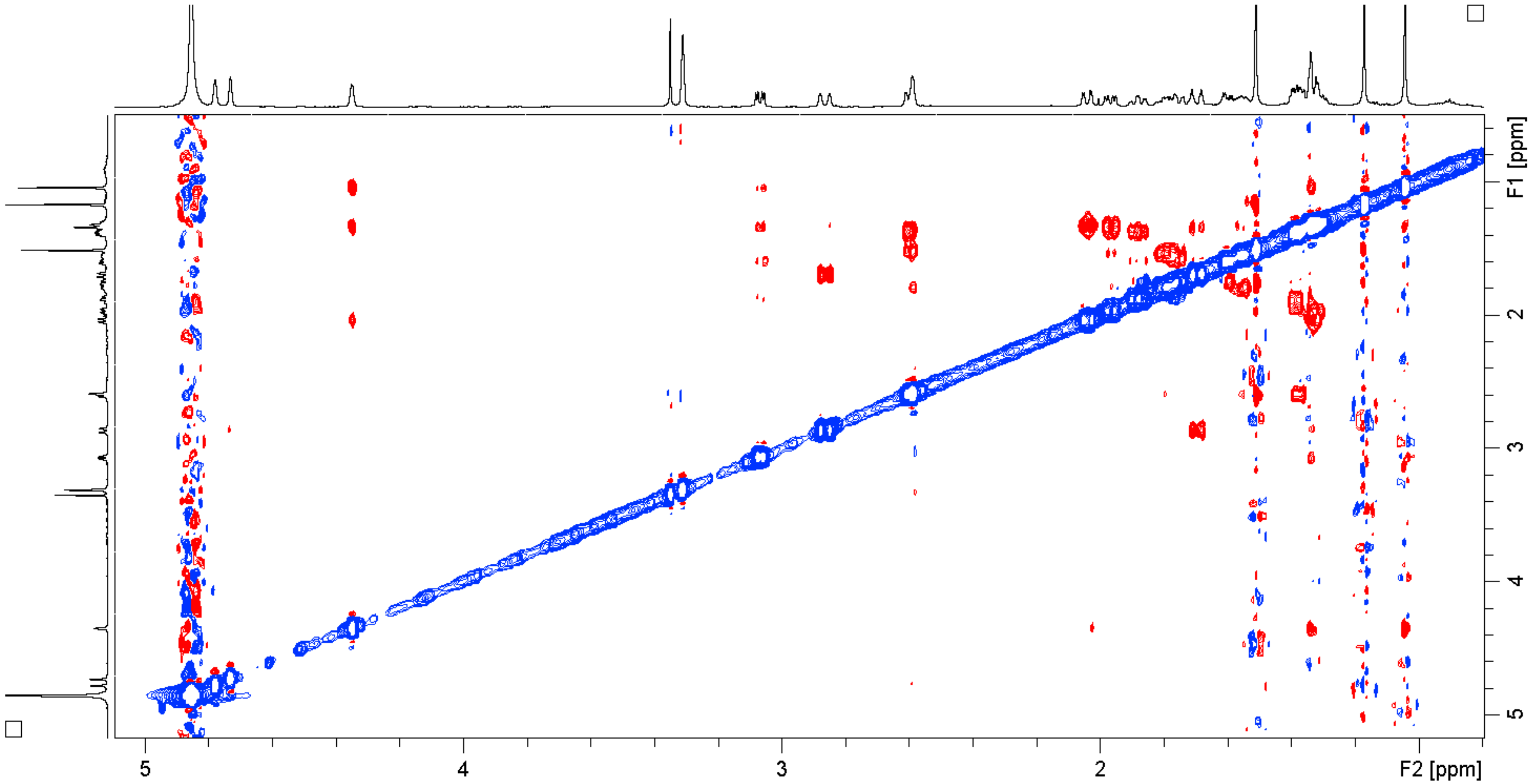


**Figure S8**. NOESY spectrum of **1** ($CD_3OD$).

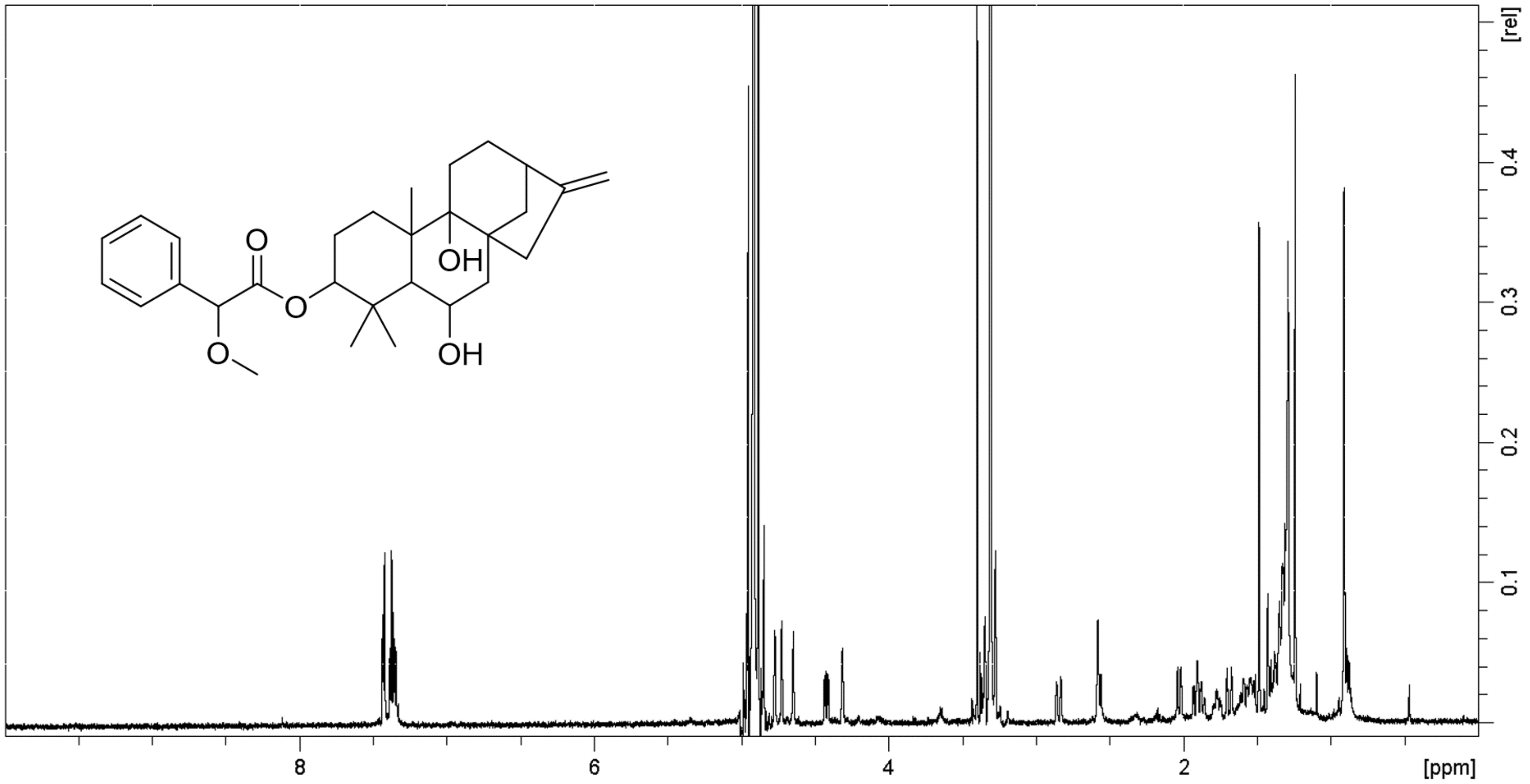


**Figure S9**. $^{1}$H NMR spectrum of (*R*)-MPA ester of **1** (**1a**) ($CD_3OD$).

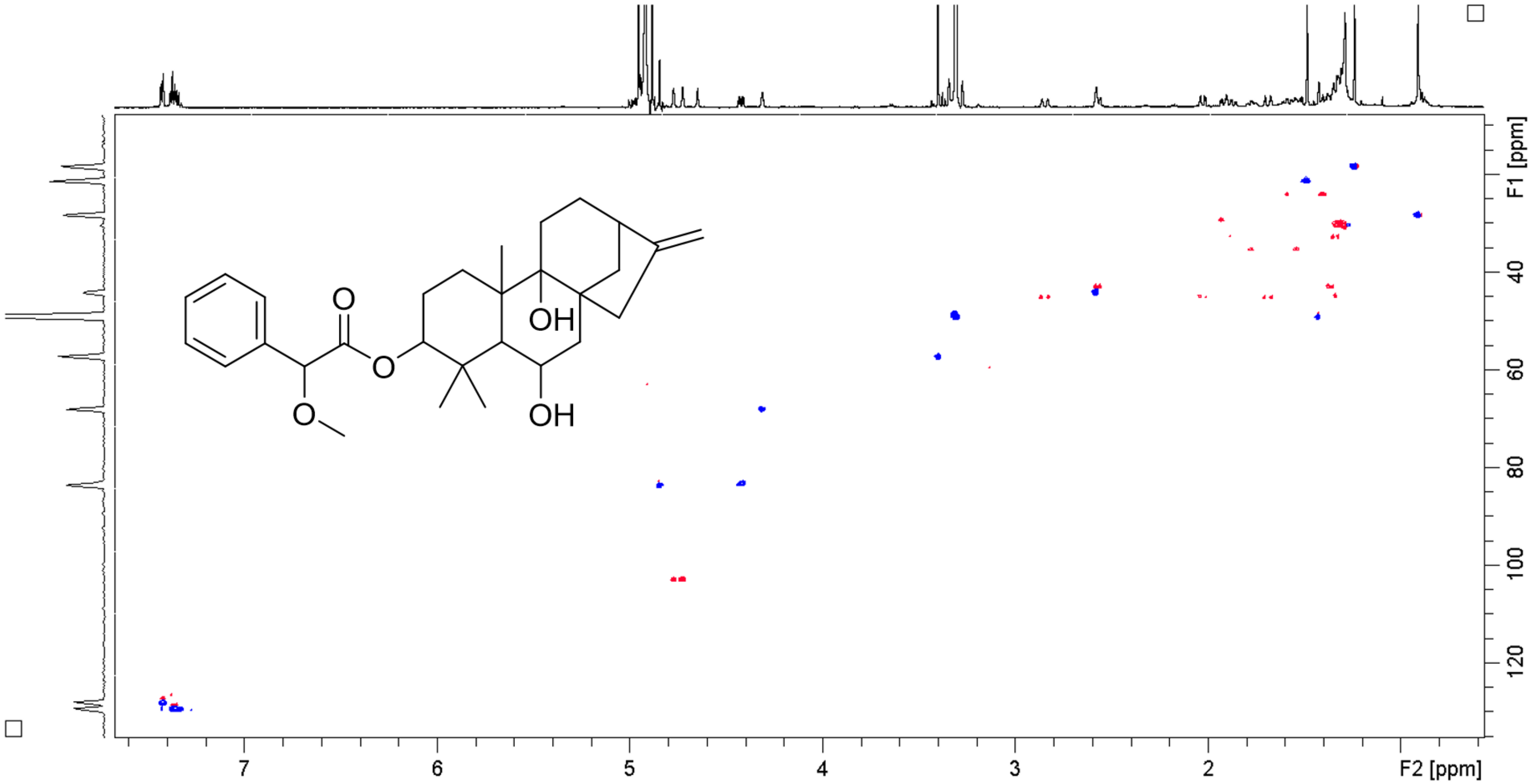


**Figure S10**. HSQC spectrum of (*R*)-MPA ester of **1** (**1a**) ($CD_3OD$).

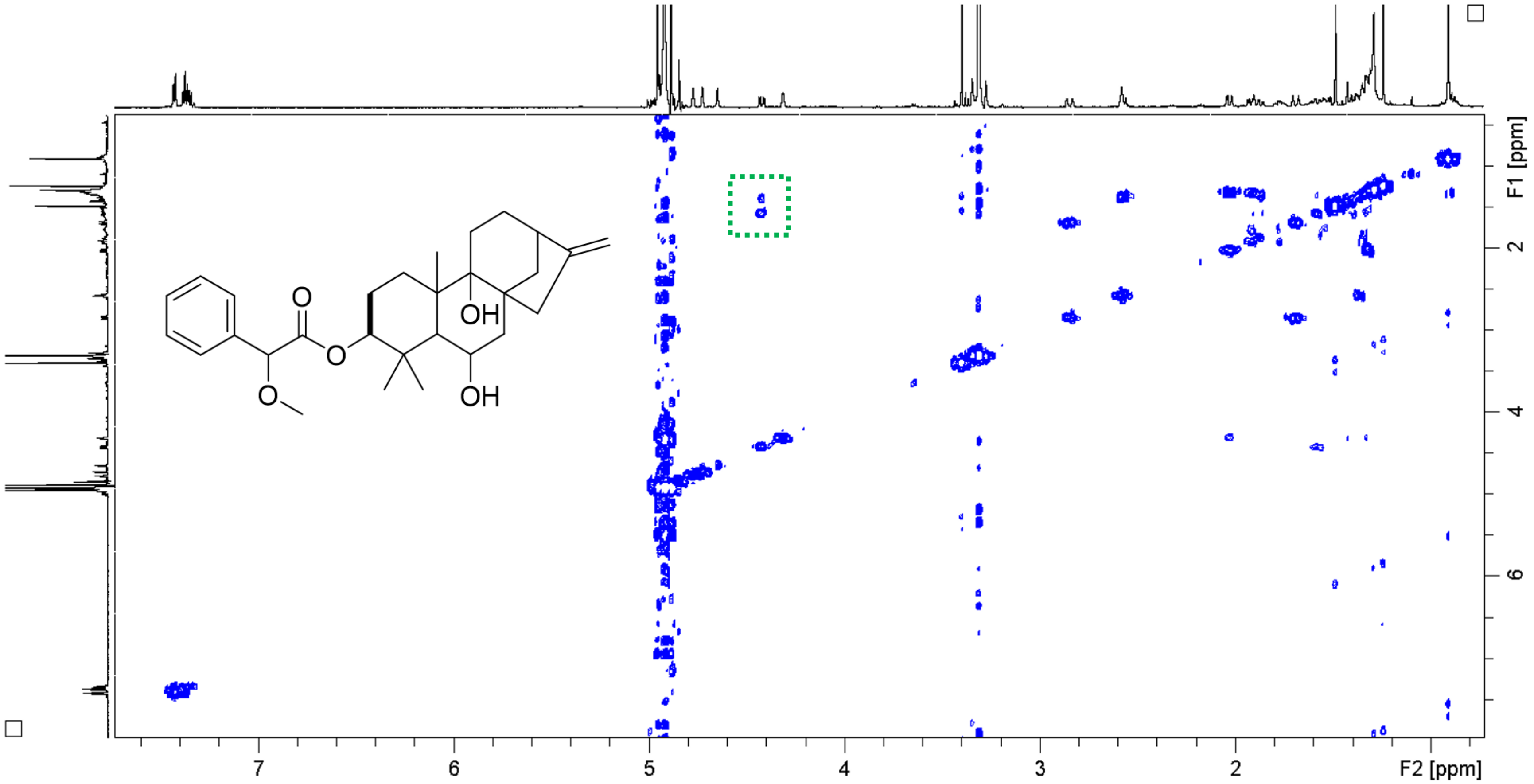


**Figure S11**. COSY spectrum of (*R*)-MPA ester of **1** (**1a**) ($CD_3OD$).

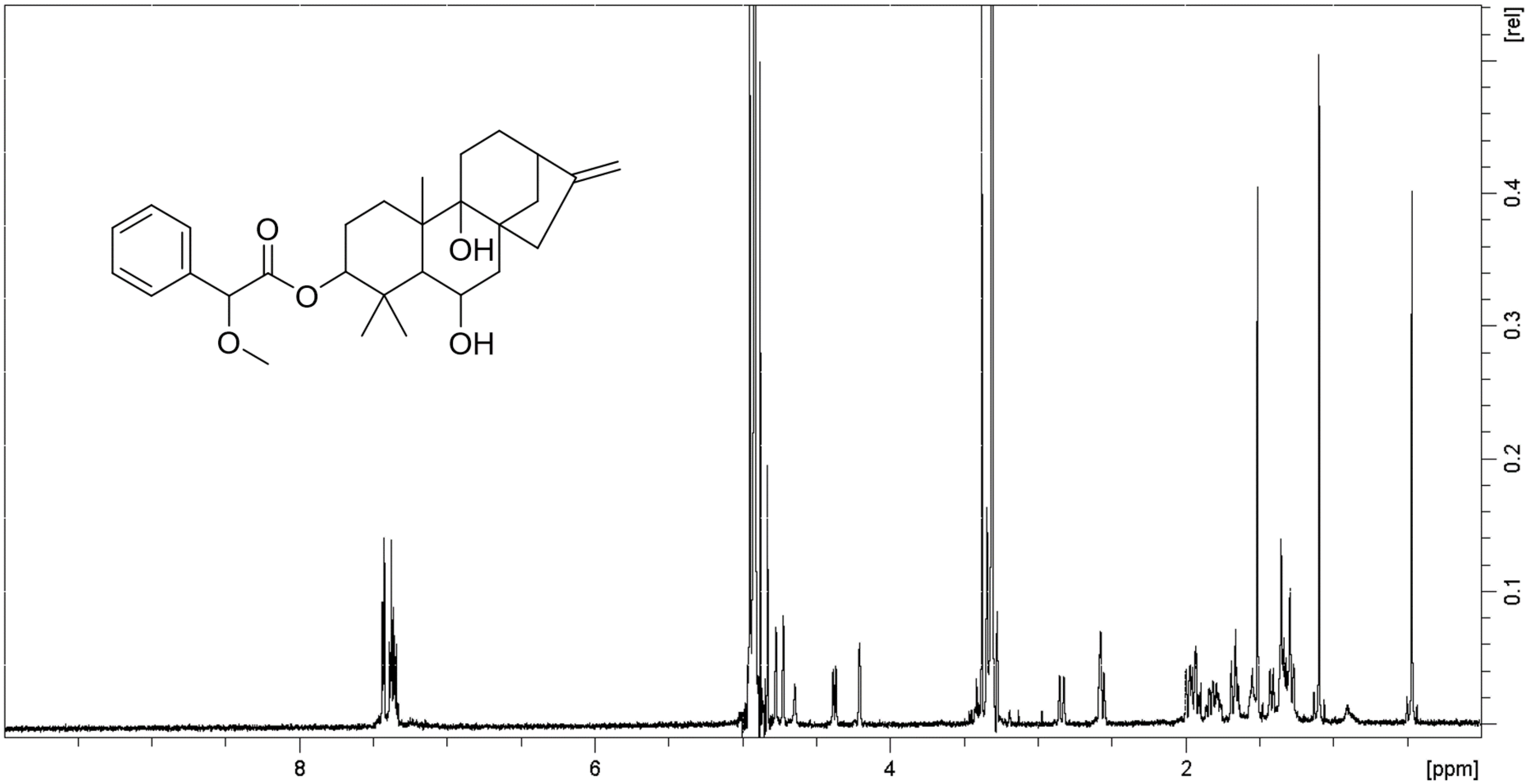


**Figure S12**. $^{1}$H NMR spectrum of (*S*)-MPA ester of **1** (**1b**) ($CD_3OD$).

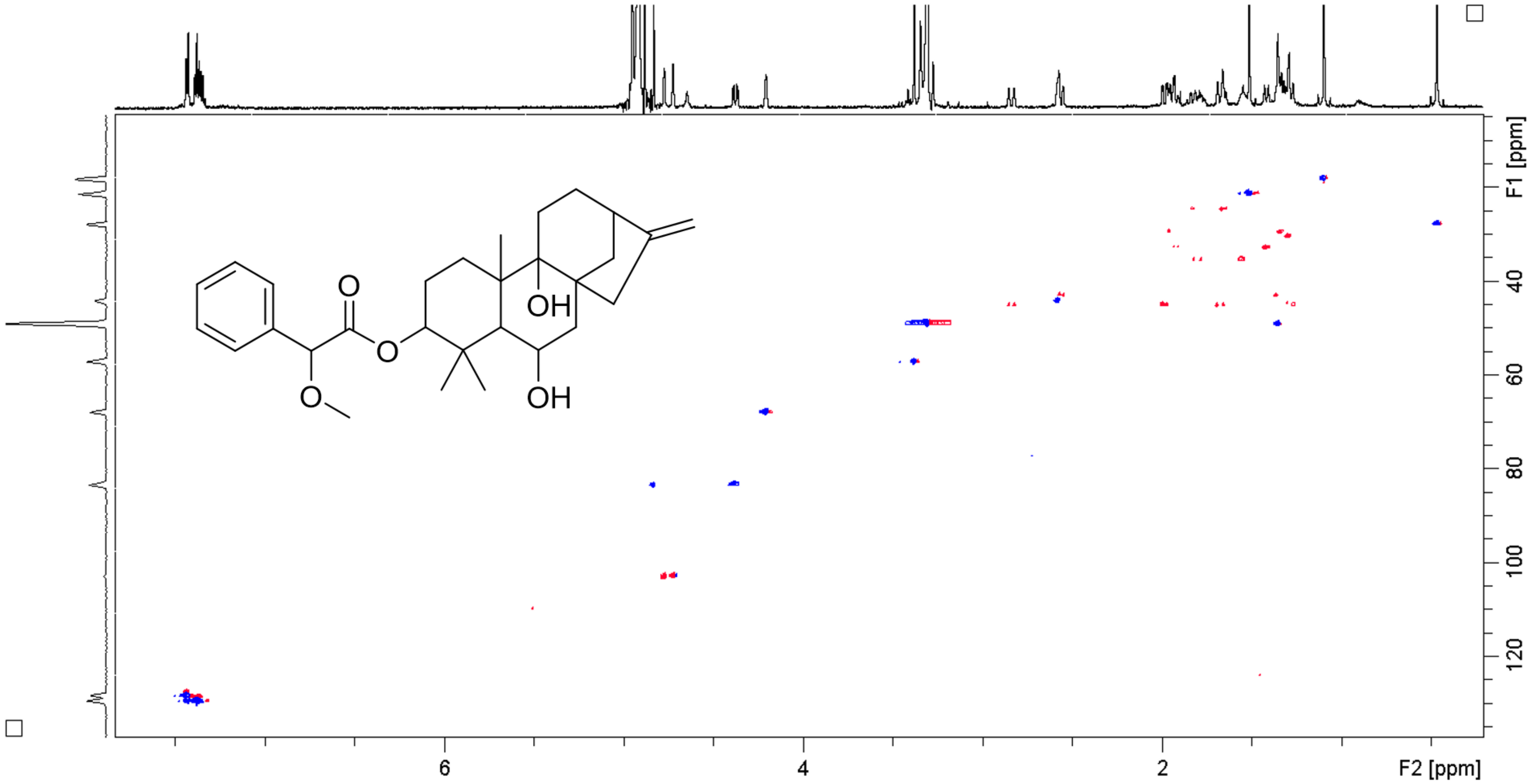


**Figure S13**. HSQC spectrum of (*S*)-MPA ester of **1** (**1b**) ($CD_3OD$).

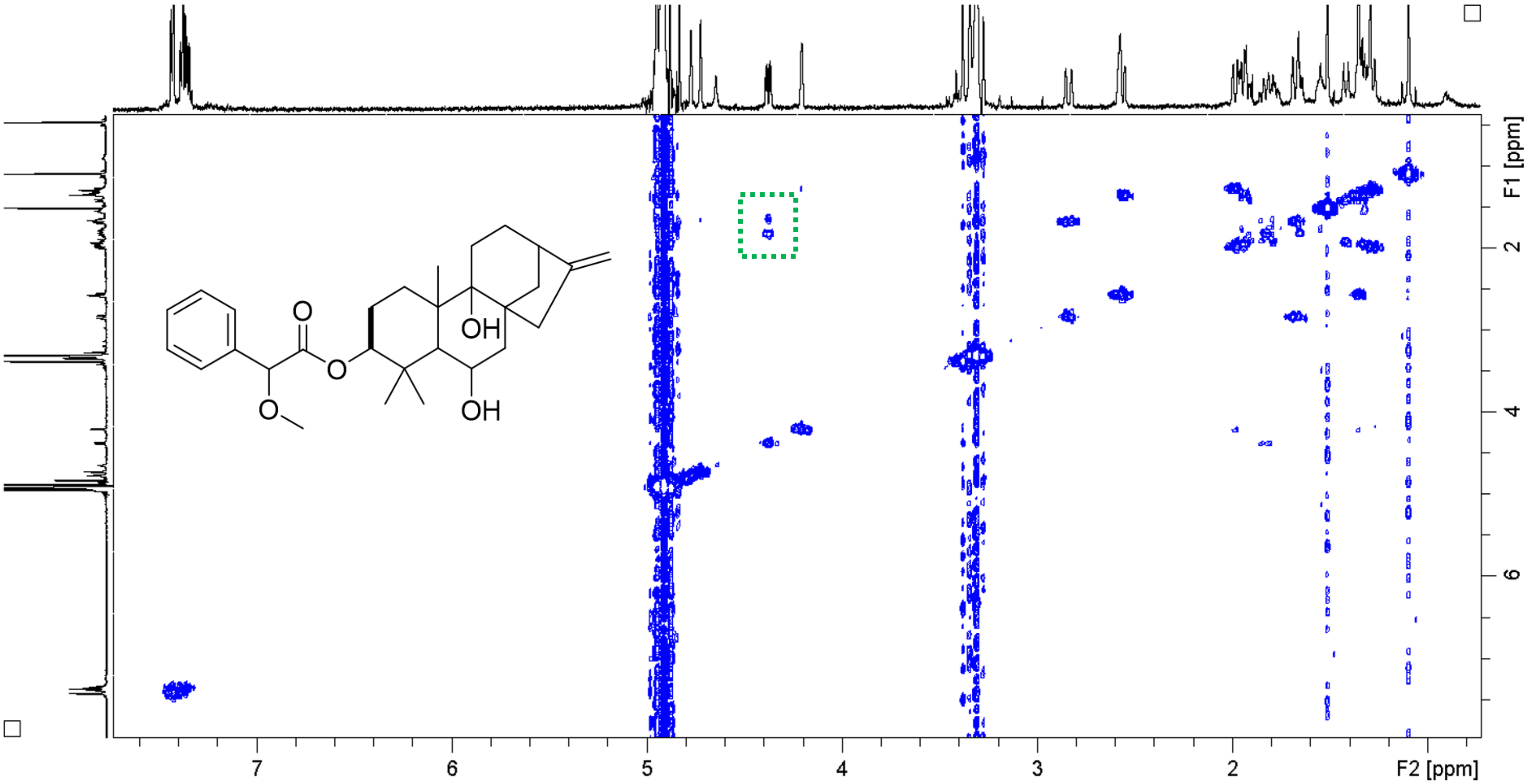


**Figure S14**. COSY spectrum of (*S*)-MPA ester of **1** (**1b**) ($CD_3OD$).

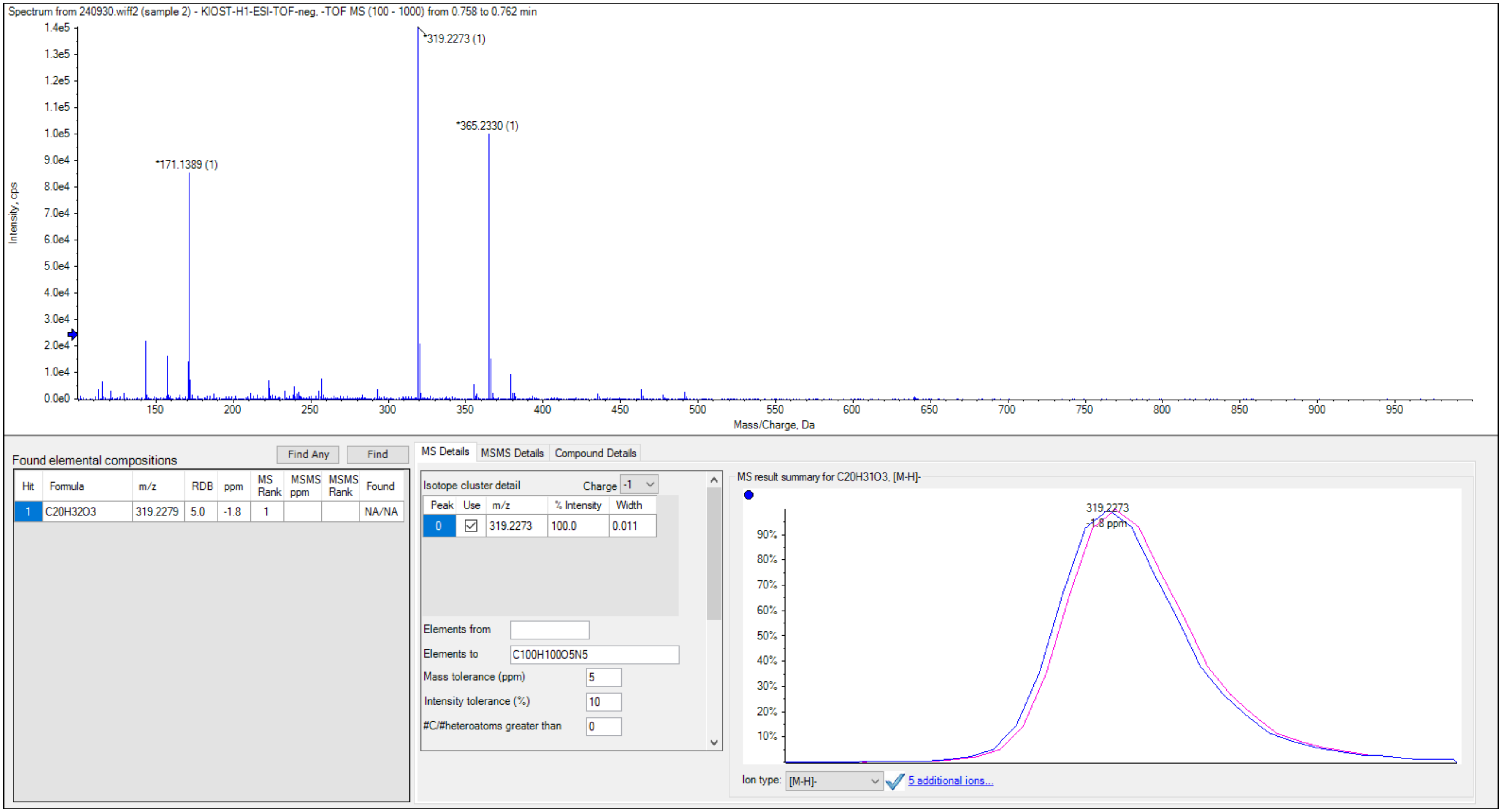


**Figure S15**. HRESIMS data of **2**.

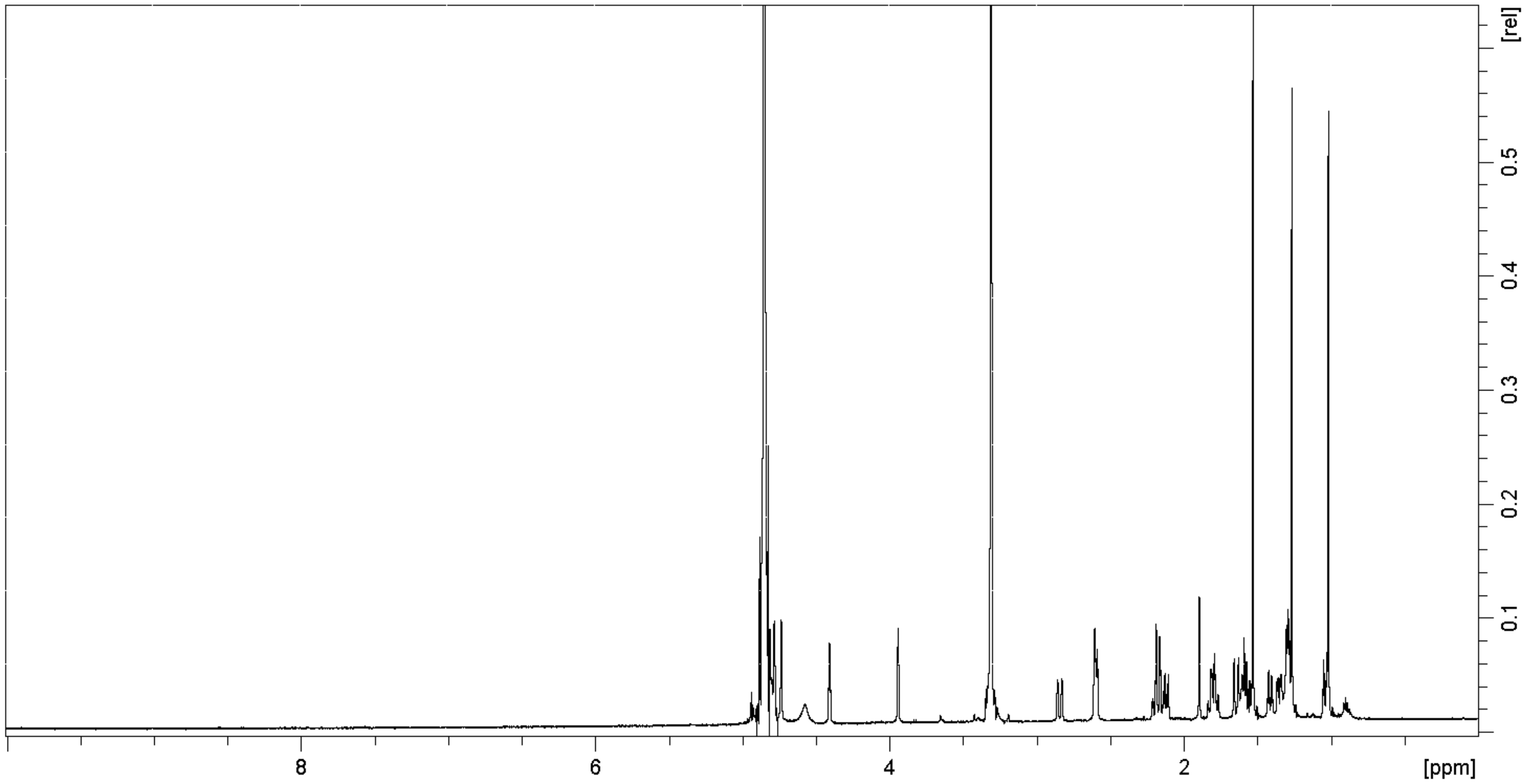


**Figure S16**. $^{1}$H NMR spectrum of **2** ($CD_3OD$).

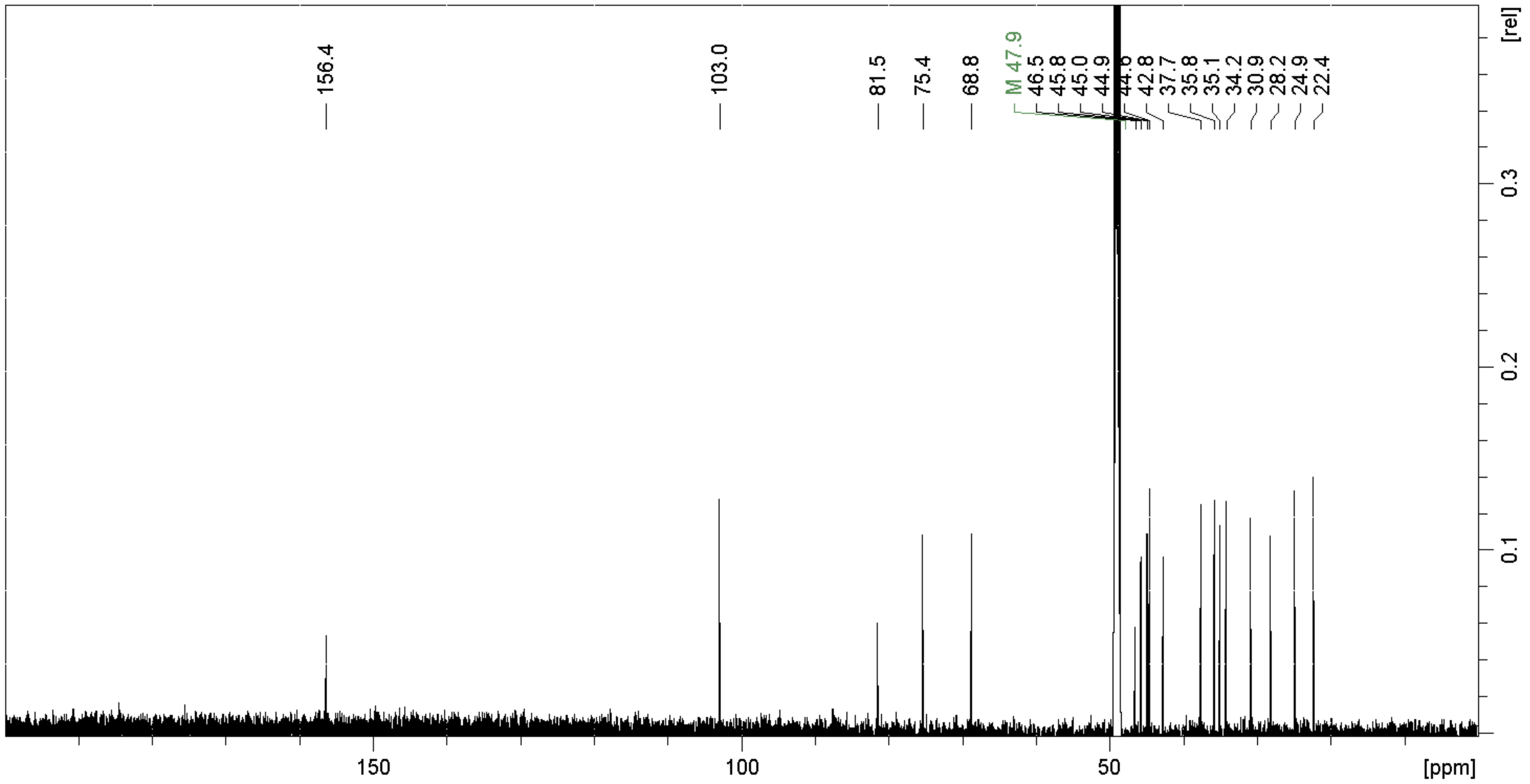


**Figure S17**. $^{13}$C NMR spectrum of **2** ($CD_3OD$).

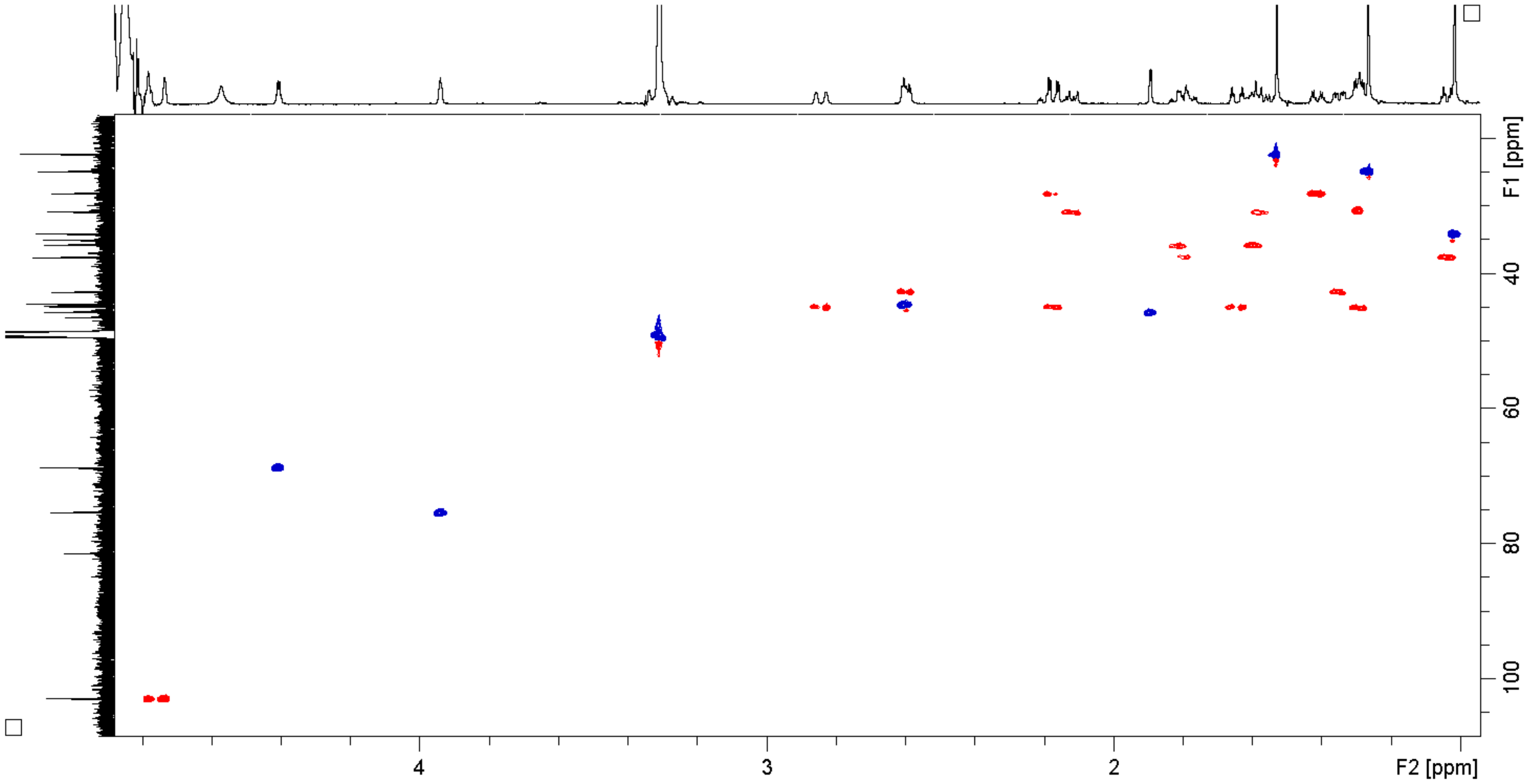


**Figure S18**. HSQC NMR spectrum of **2** ($CD_3OD$).

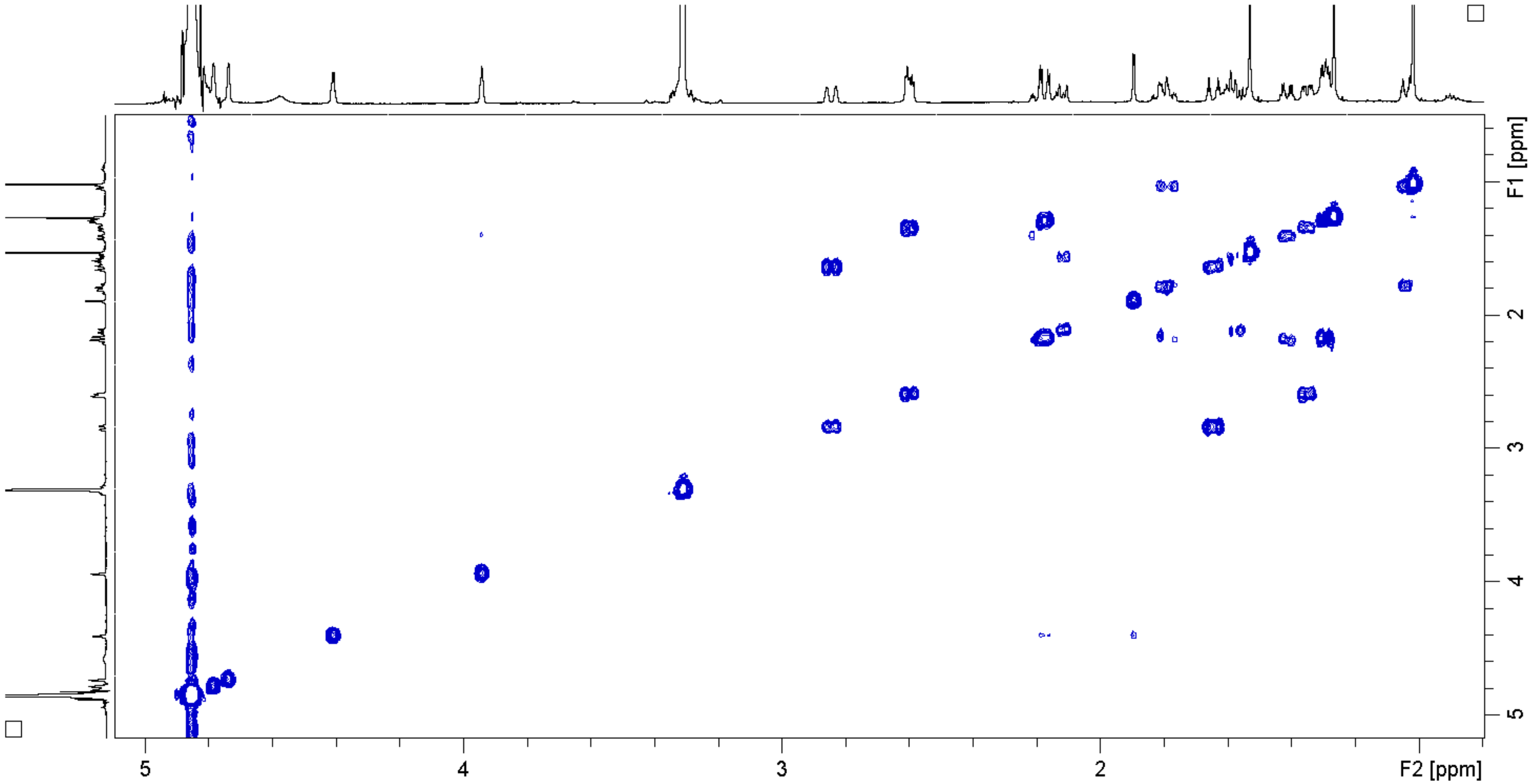


**Figure S19**. $^{1}H$-$^{1}H$ COSY NMR spectrum of **2** ($CD_3OD$).

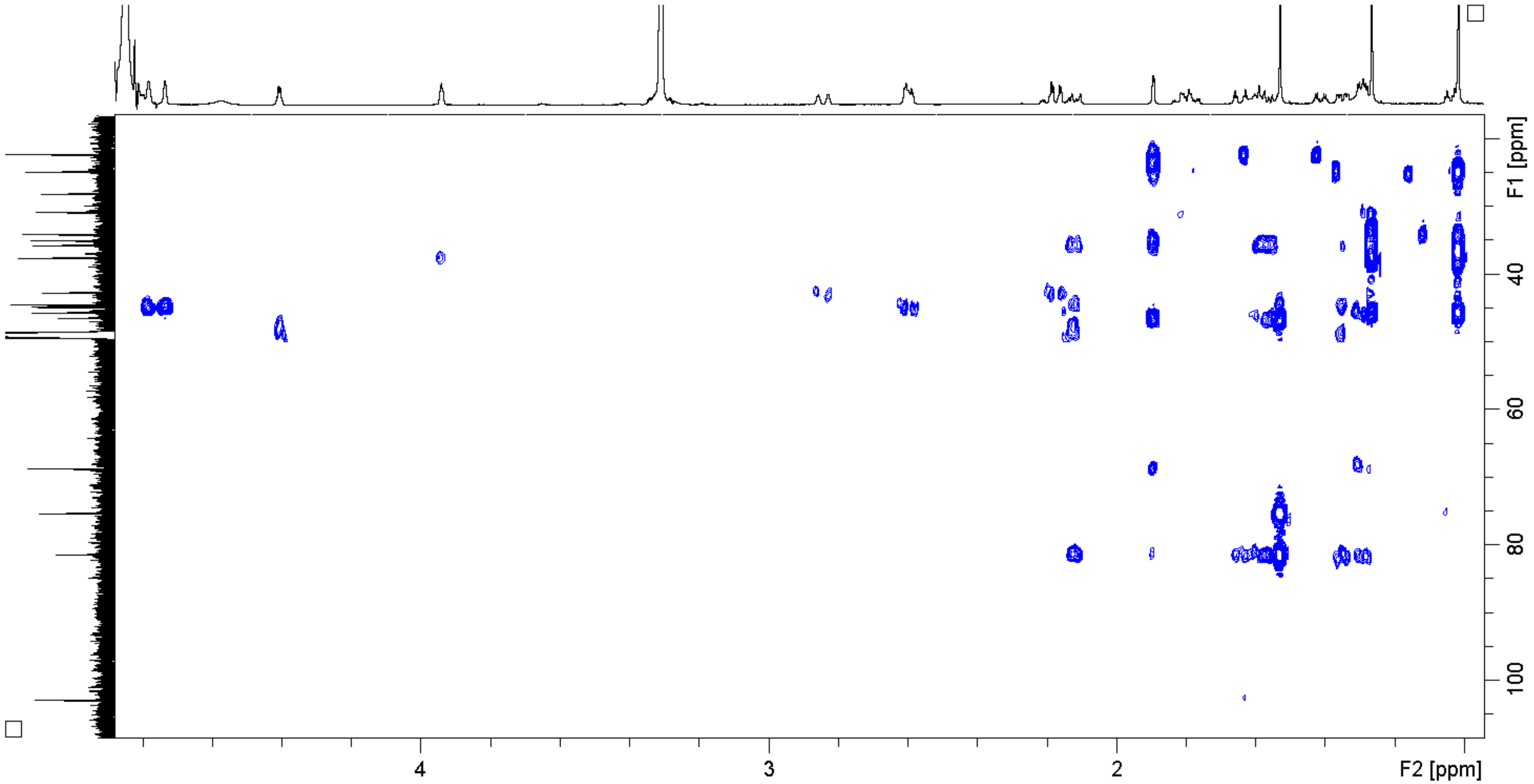


**Figure S20**. HMBC NMR spectrum of **2** ($CD_3OD$).

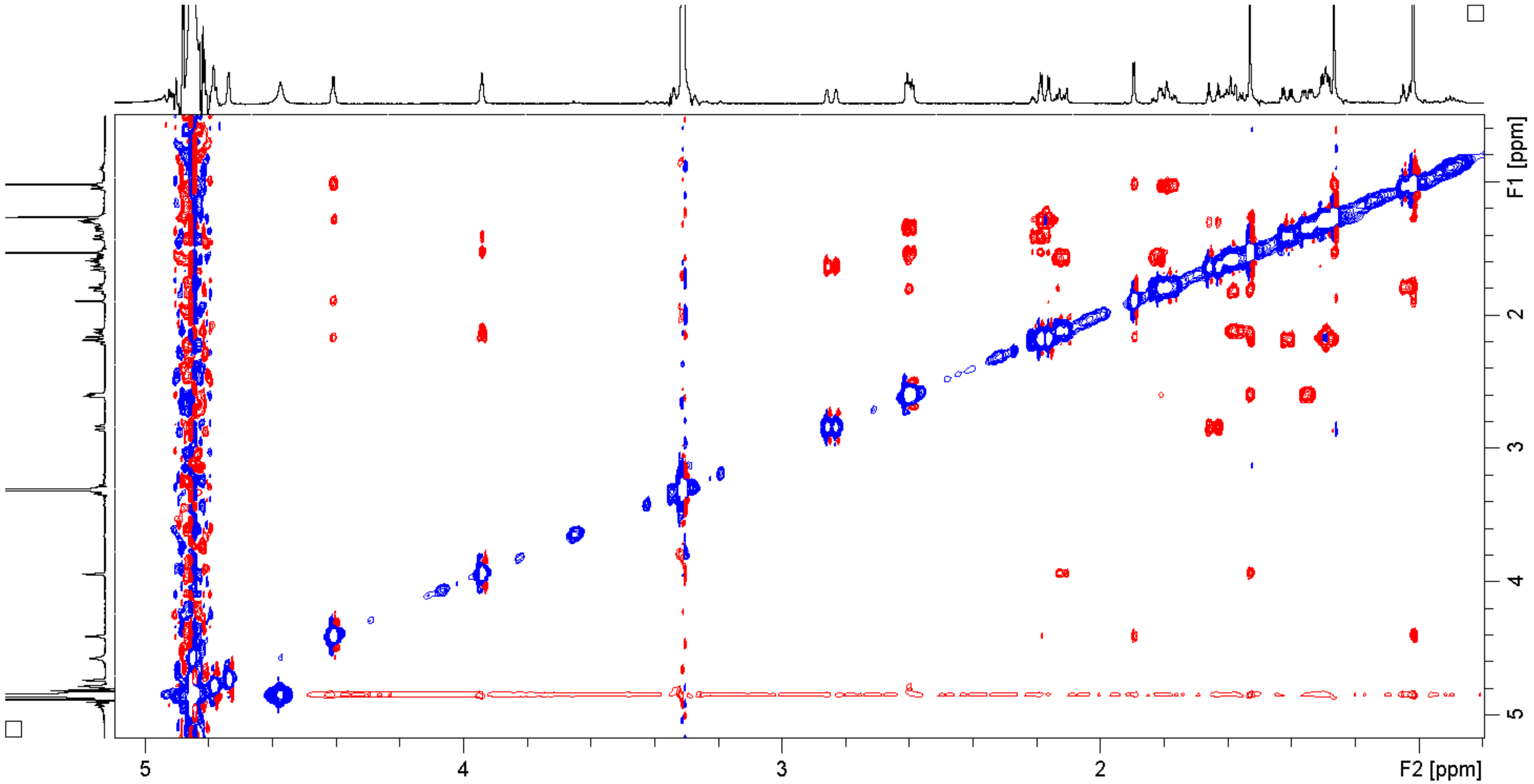


**Figure S21**. NOESY NMR spectrum of **2** ($CD_3OD$).

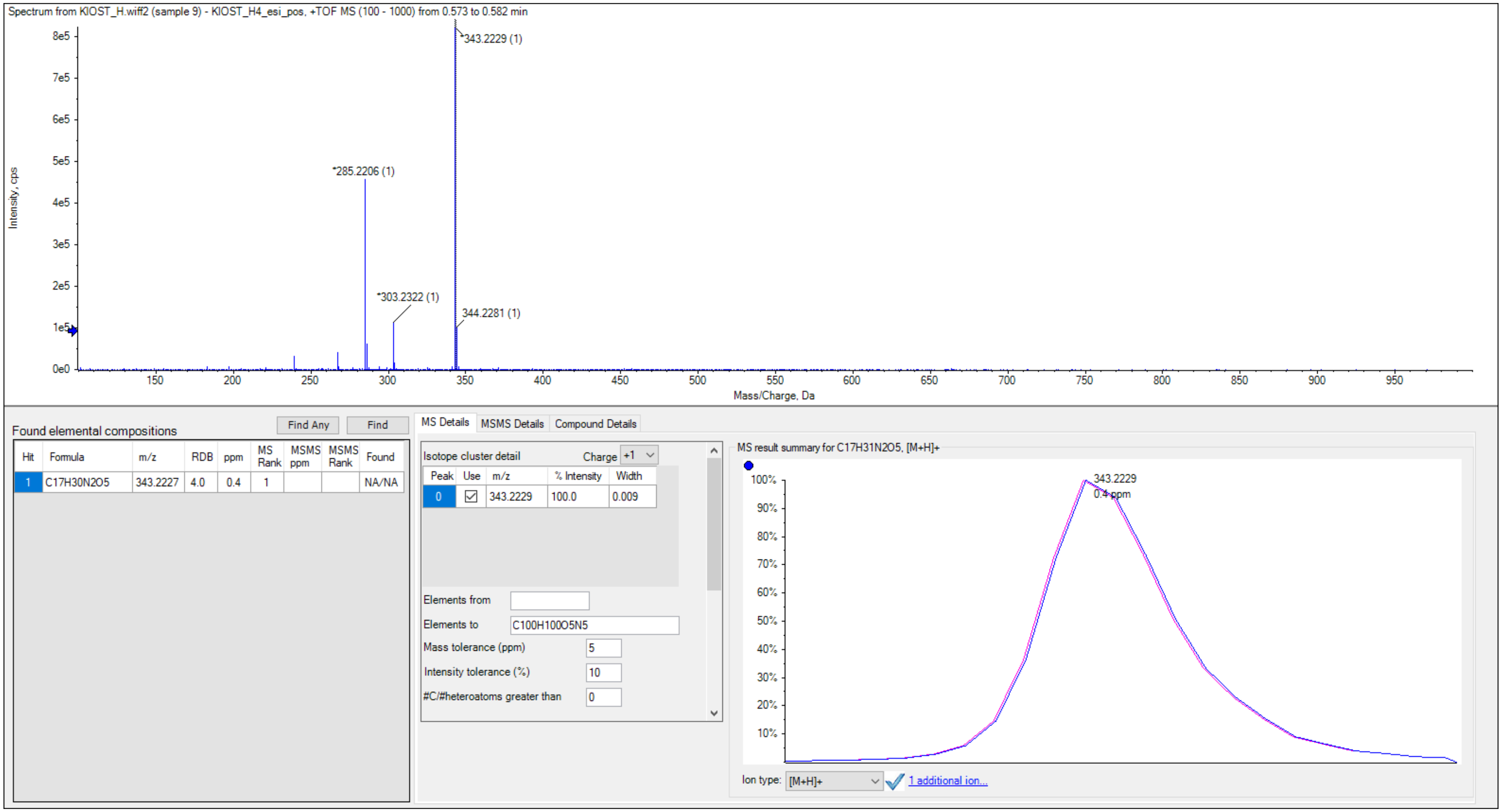


**Figure S22**. HRESIMS data of **3**.

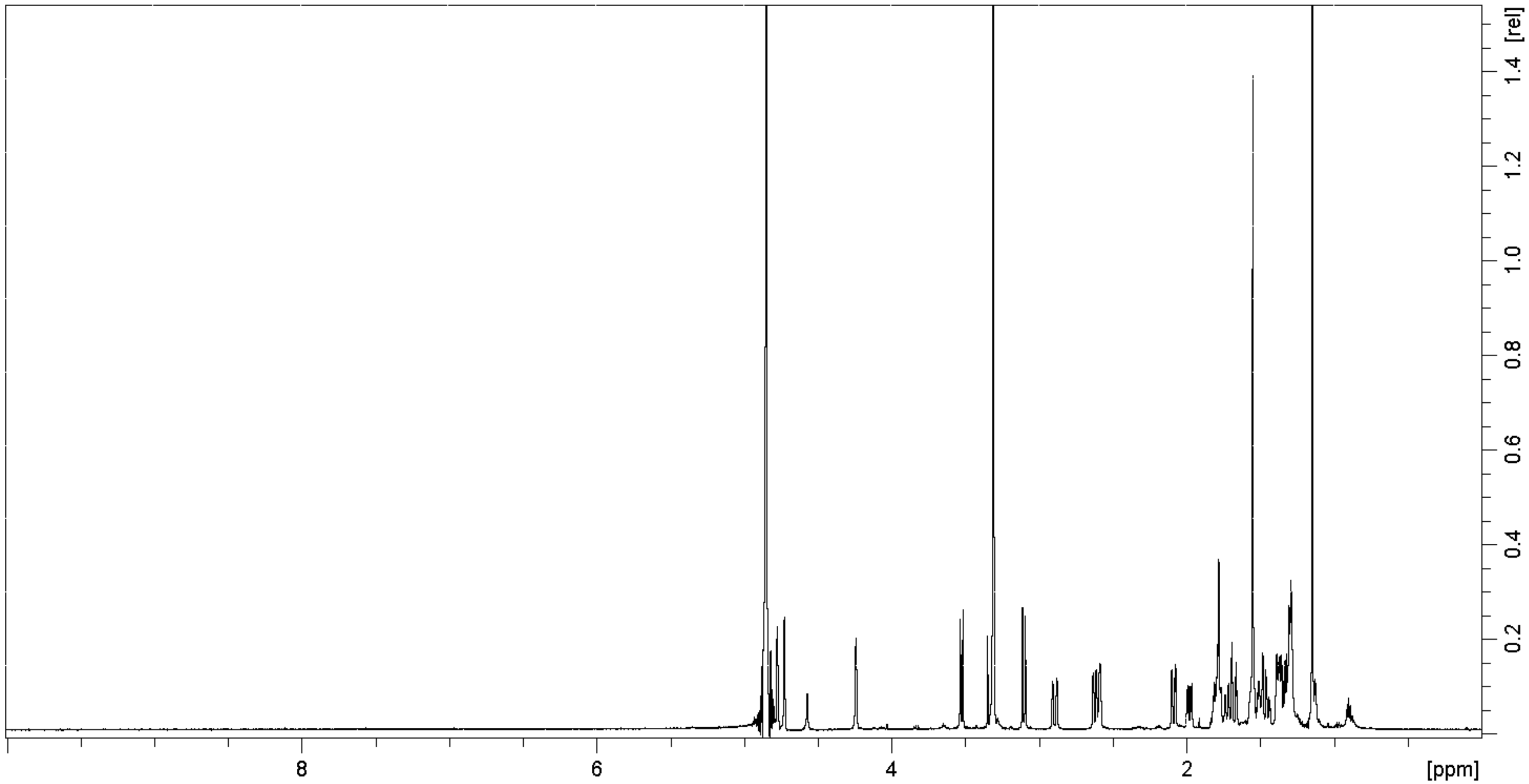


**Figure S23**. $^{1}$H NMR spectrum of **3** ($CD_3OD$).

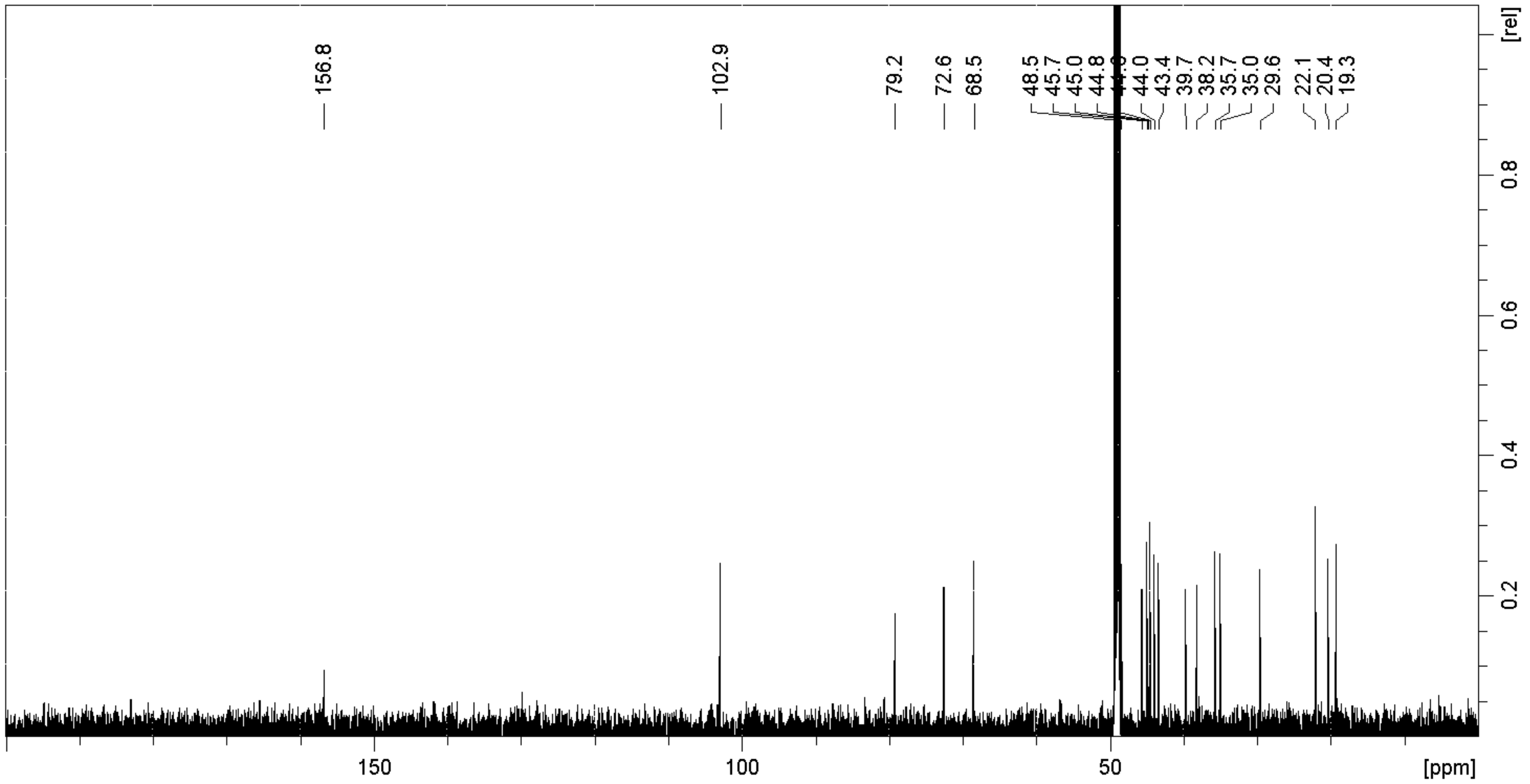


**Figure S24**. $^{13}C$ NMR spectrum of **3** ($CD_3OD$).

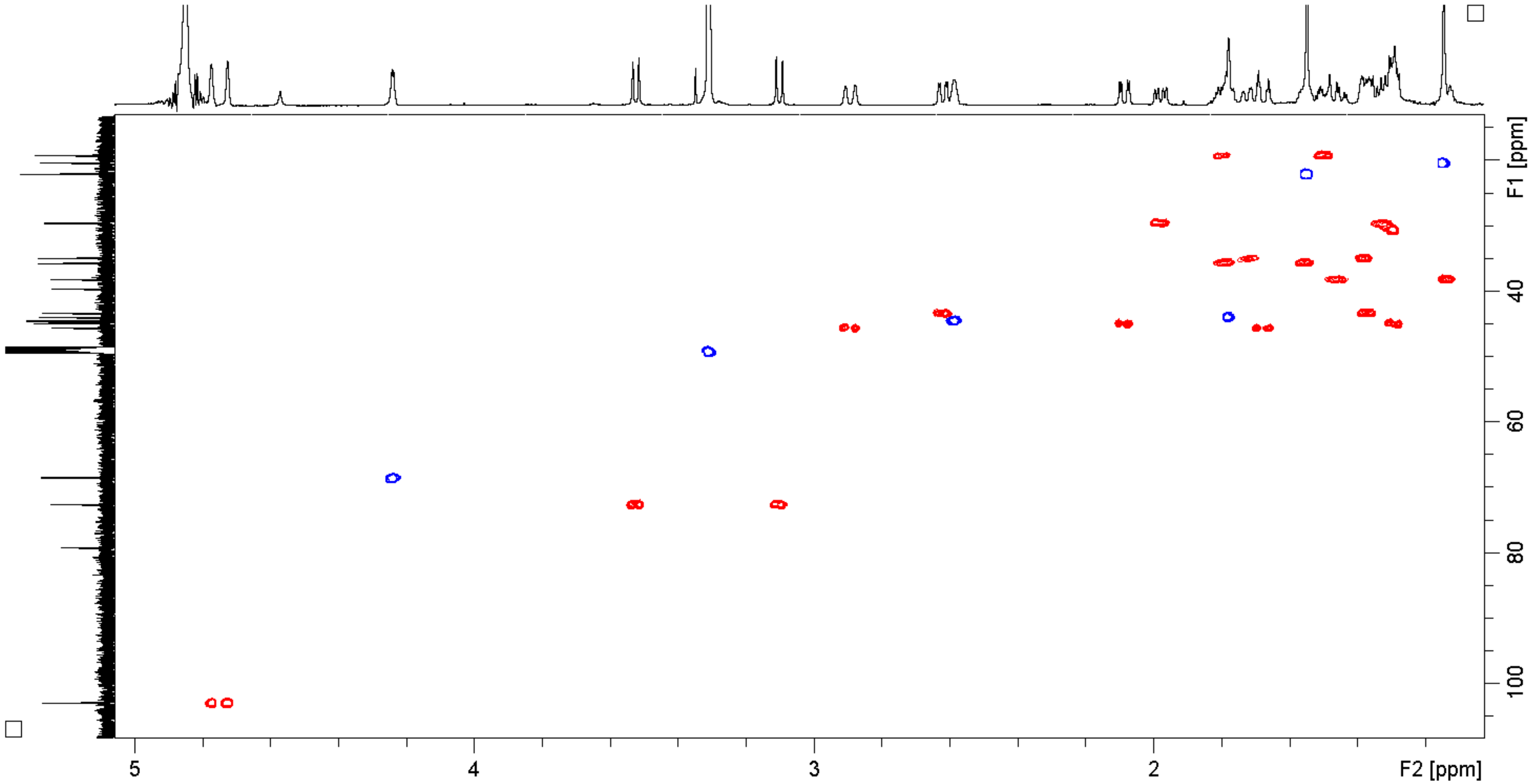


**Figure S25**. HSQC NMR spectrum of **3** ($CD_3OD$).

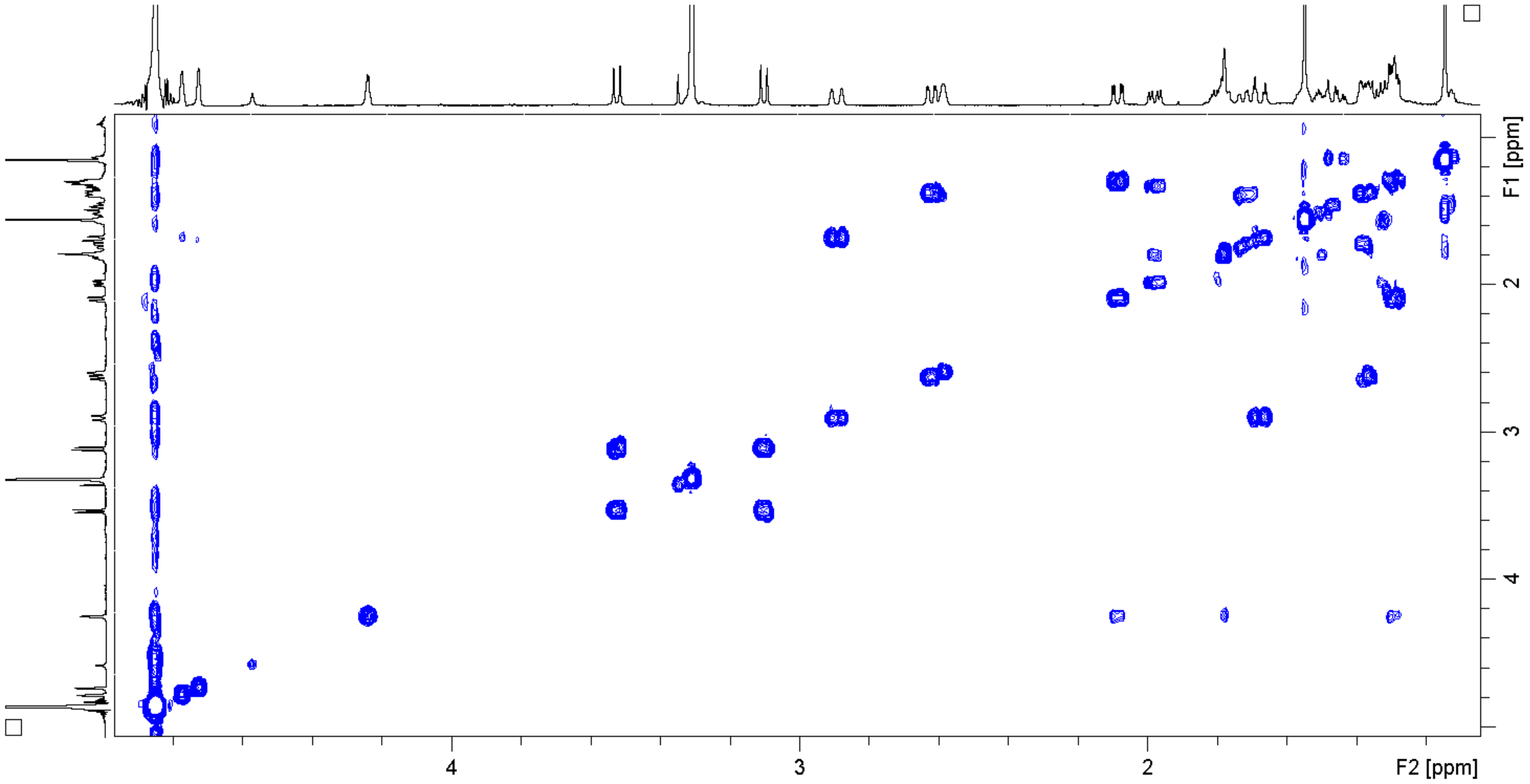


**Figure S26**. $^{1}$H-$^{1}$H COSY NMR spectrum of **3** ($CD_3OD$).

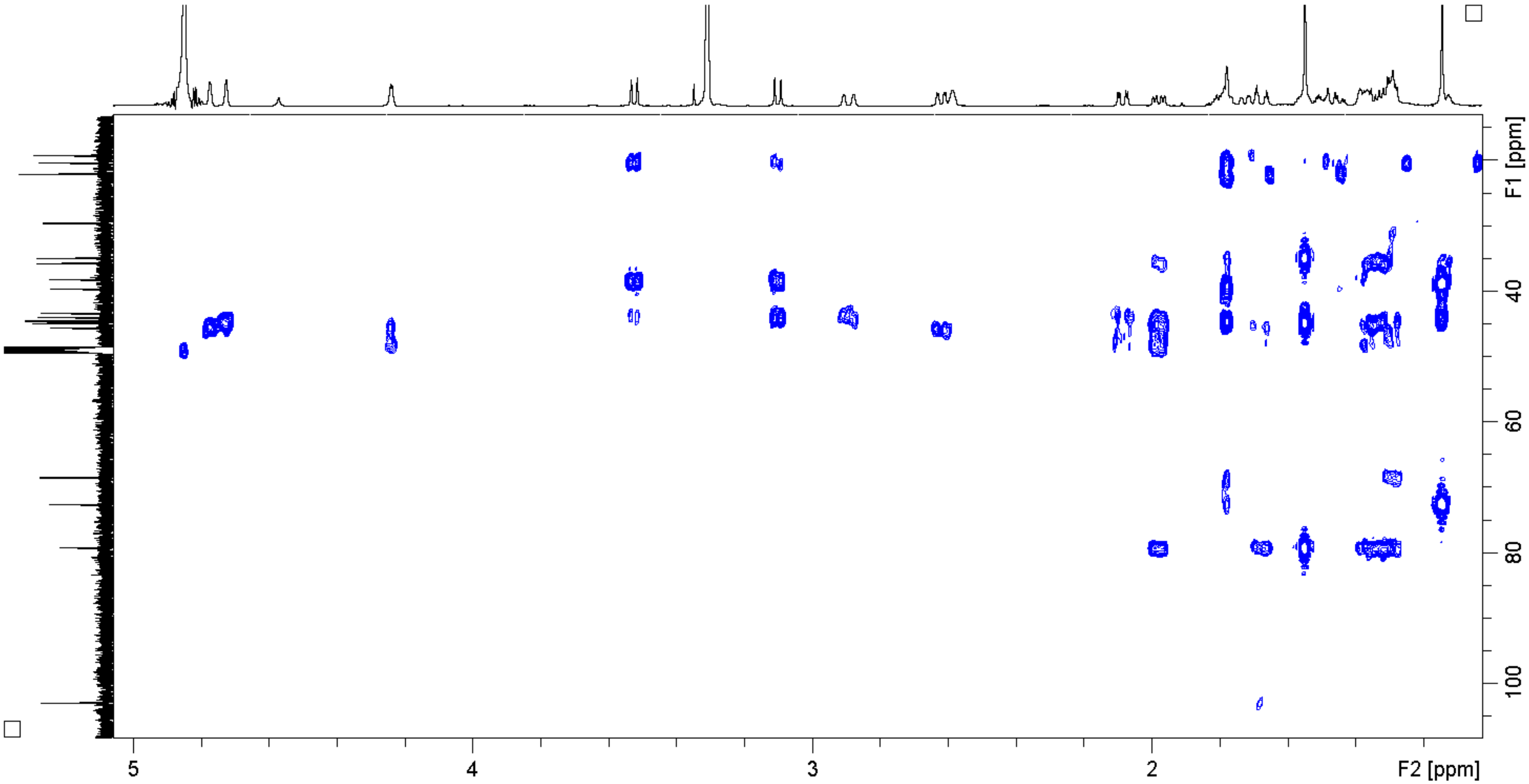


**Figure S27**. HMBC NMR spectrum of **3** ($CD_3OD$).

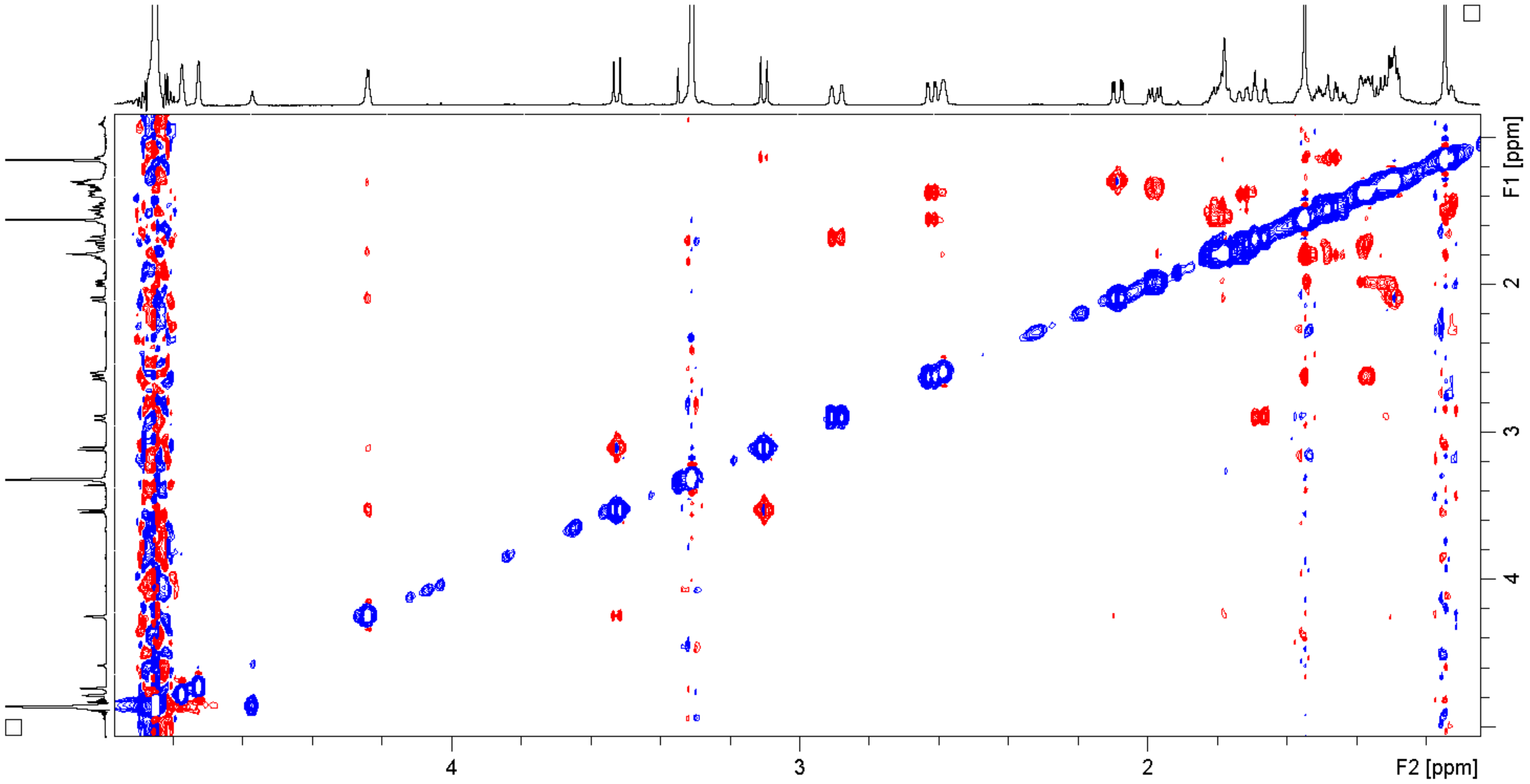


**Figure S28**. NOESY NMR spectrum of **3** ($CD_3OD$).

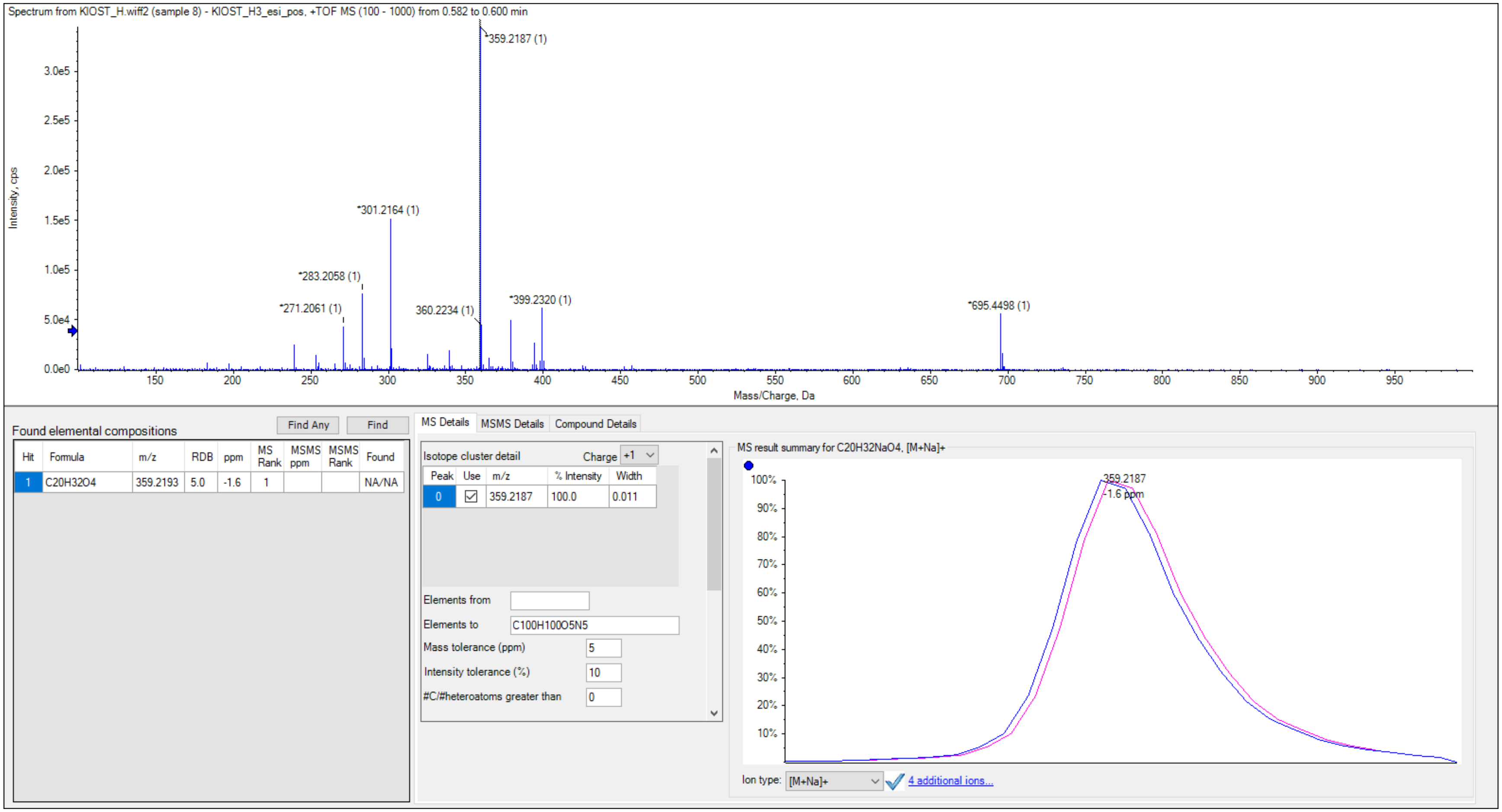


**Figure S29**. HRESIMS data of **4**.

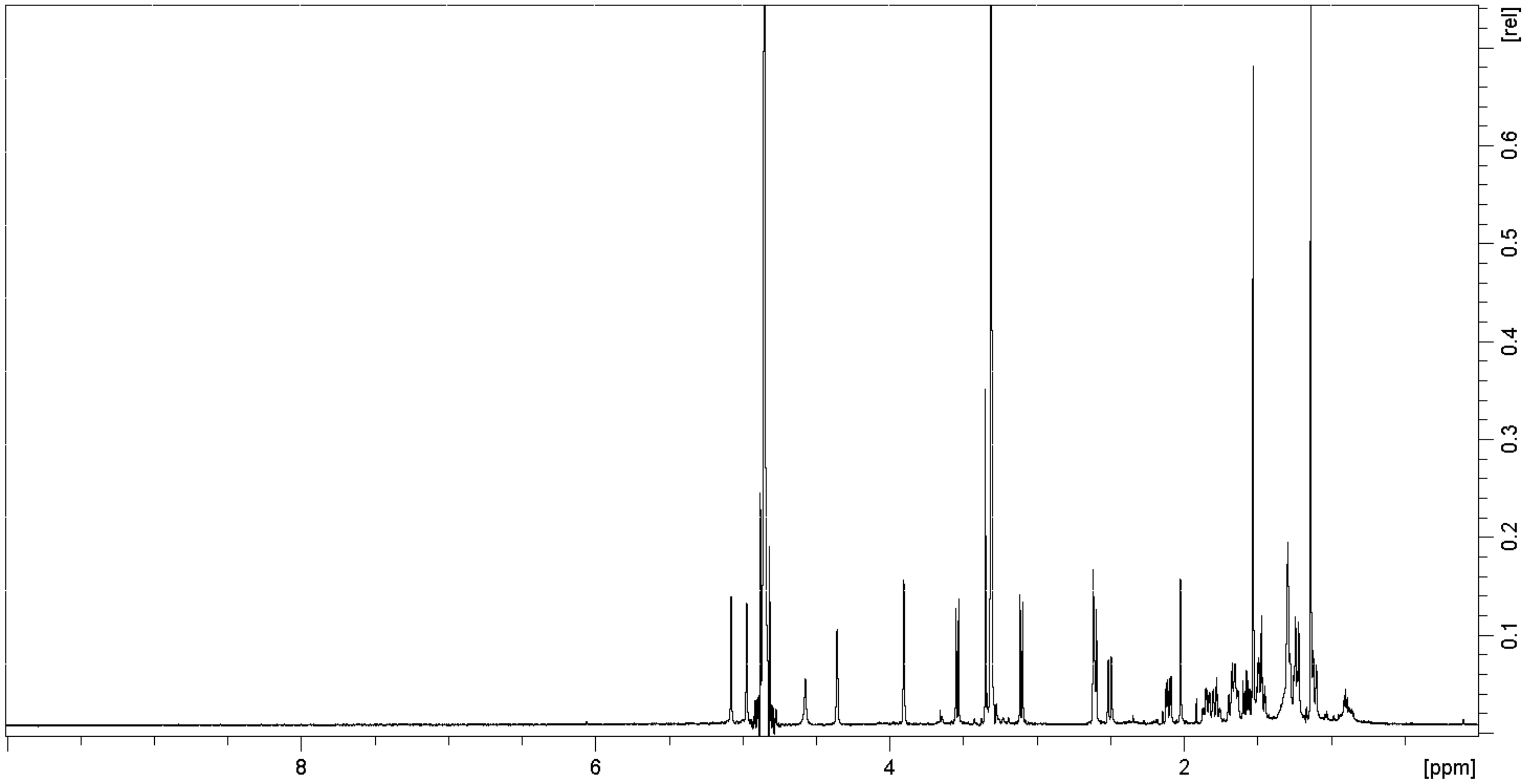


**Figure S30**. $^{1}$H NMR spectrum of **4** ($CD_3OD$).

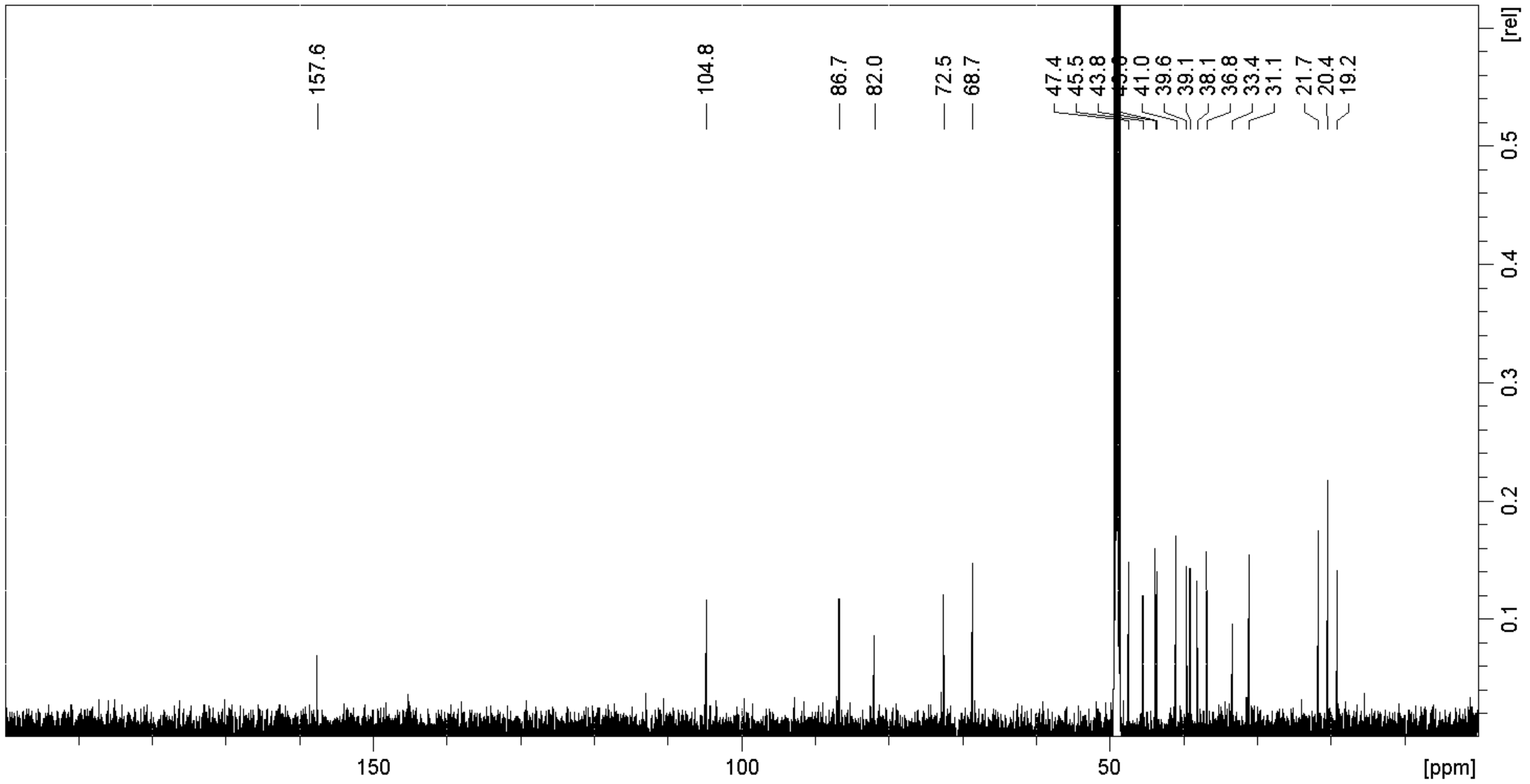


**Figure S31**. $^{13}$C NMR spectrum of **4** ($CD_3OD$).

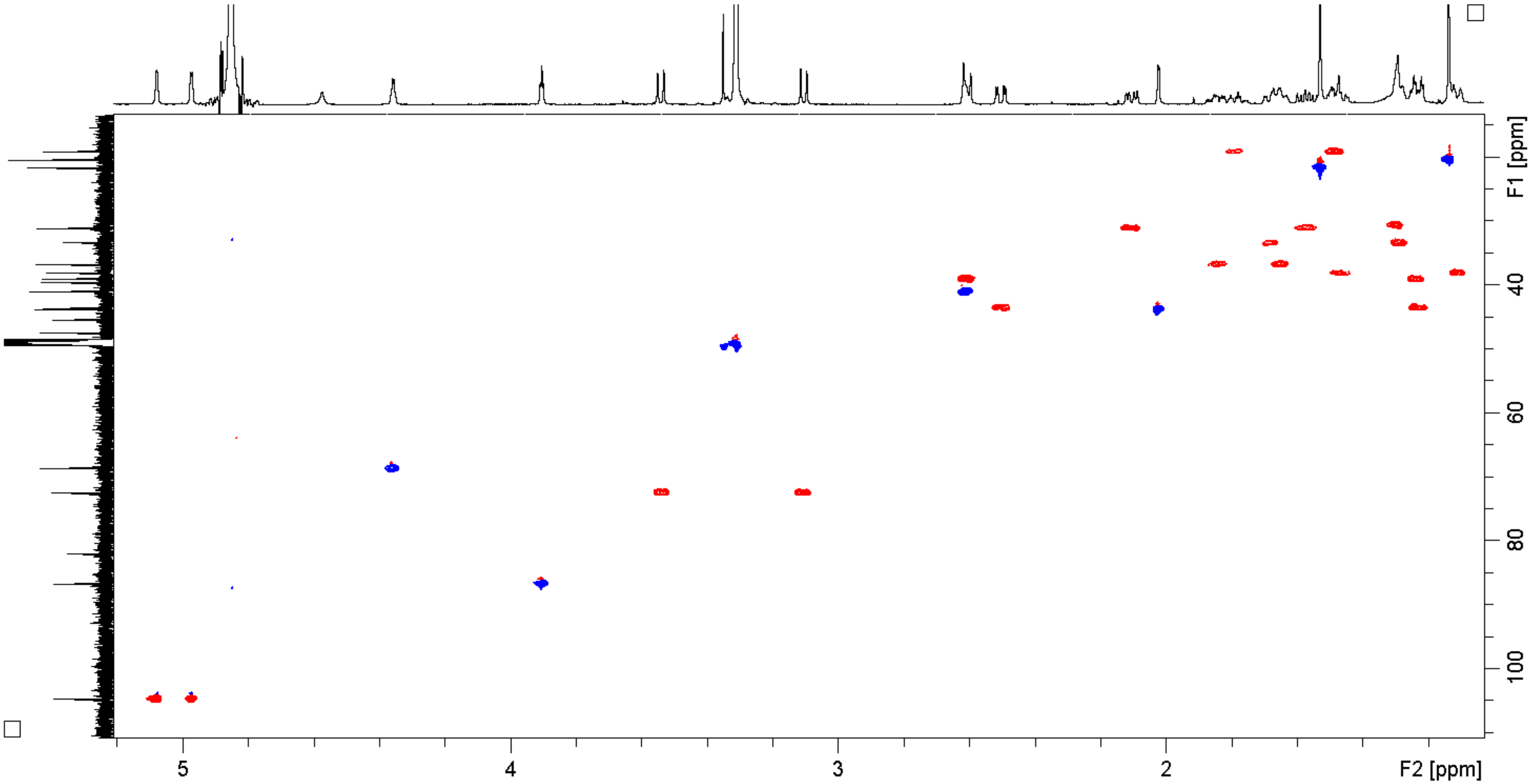


**Figure S32**. HSQC NMR spectrum of **4** ($CD_3OD$).

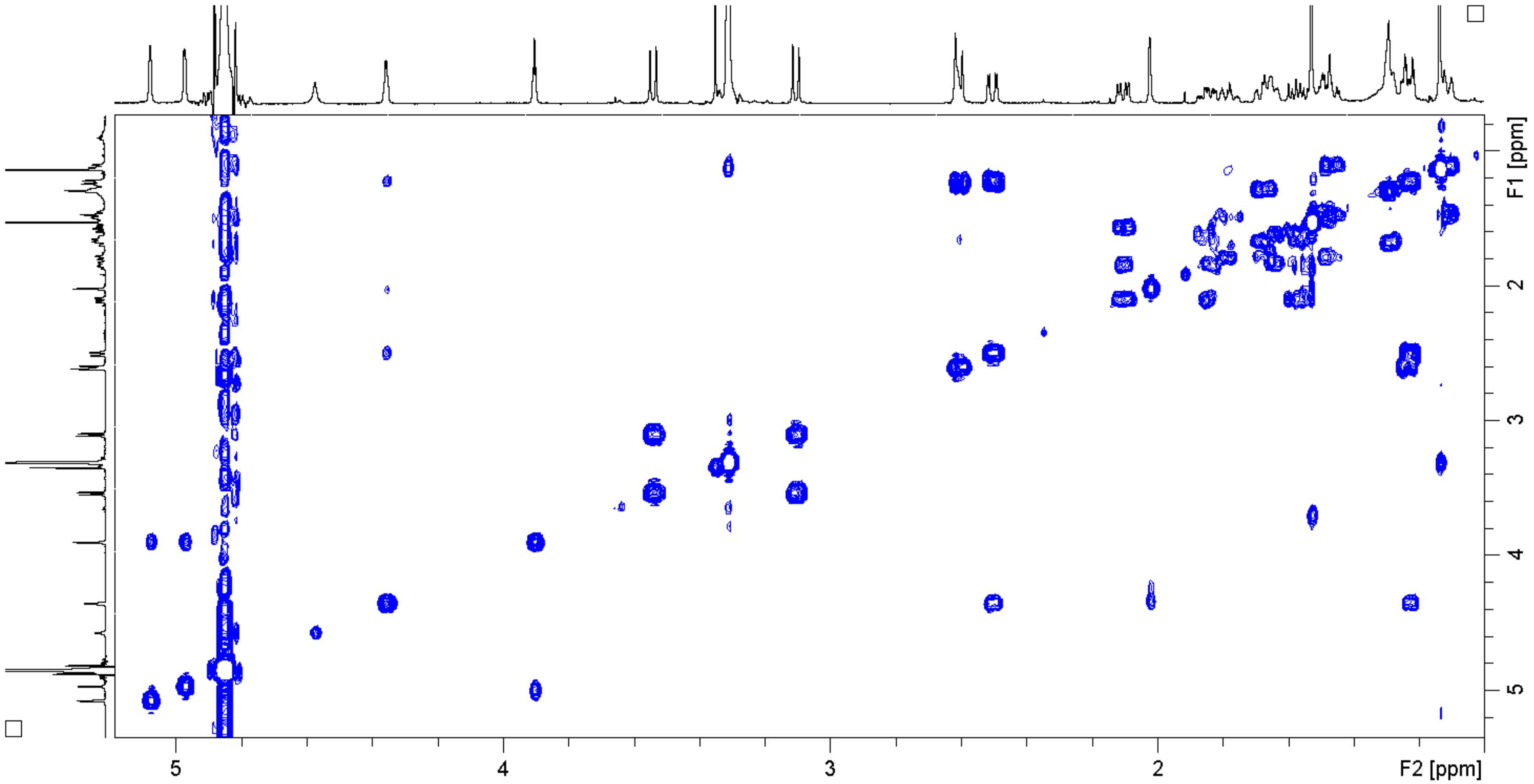


**Figure S33**. $^1$H-$^1$H COSY NMR spectrum of **4** ($CD_3OD$).

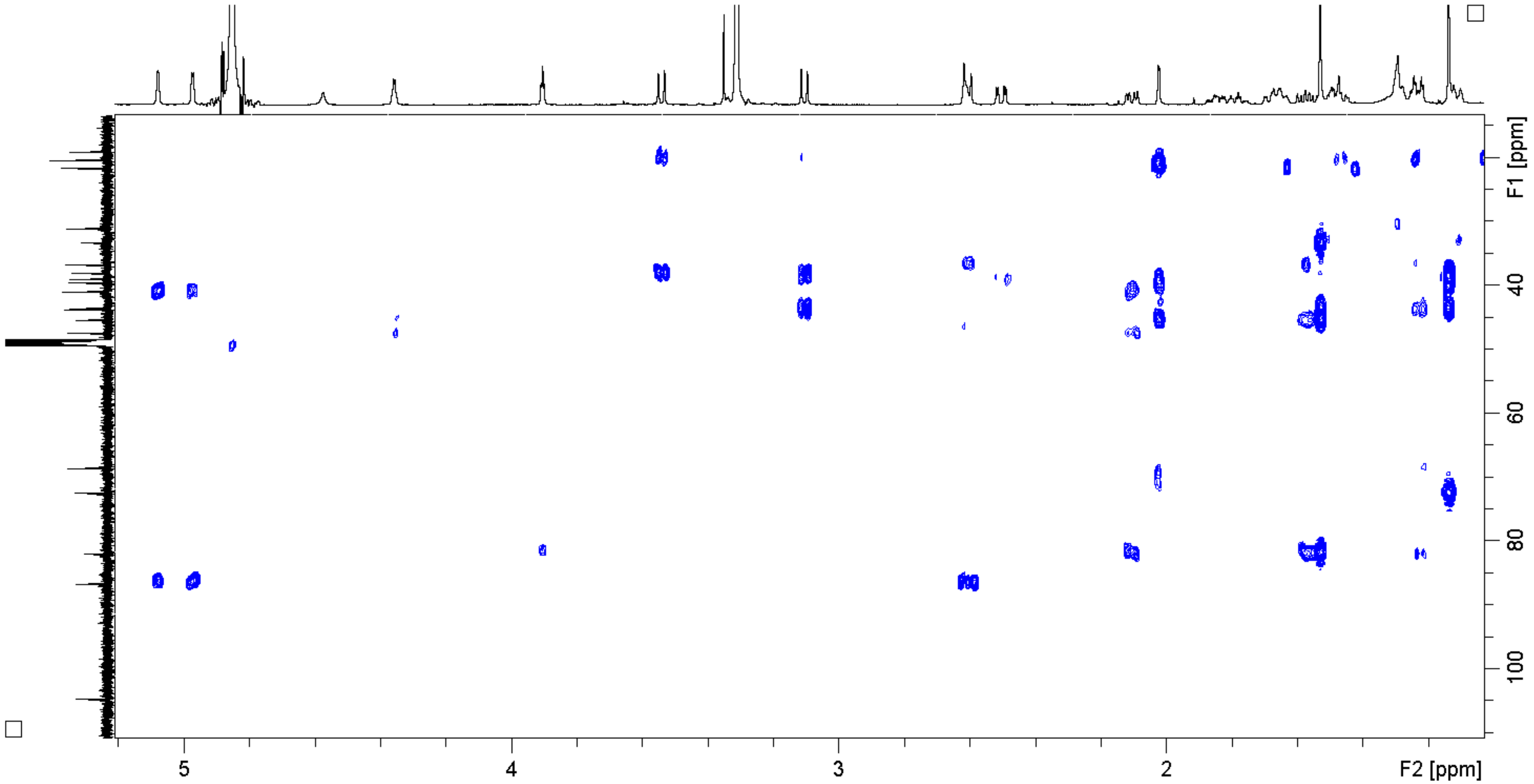


**Figure S34**. HMBC NMR spectrum of **4** ($CD_3OD$).

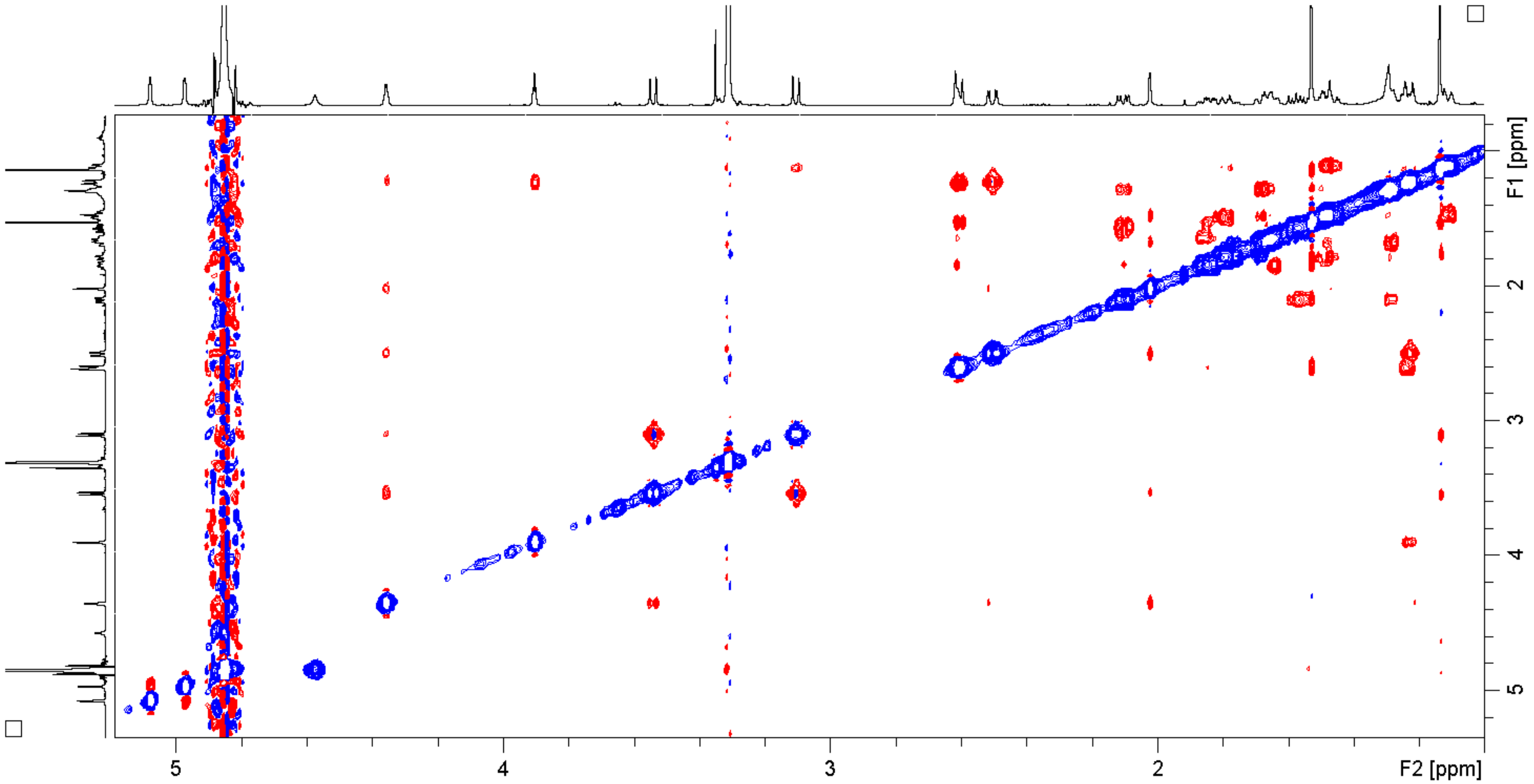


**Figure S35**. NOESY NMR spectrum of **4** ($CD_3OD$).

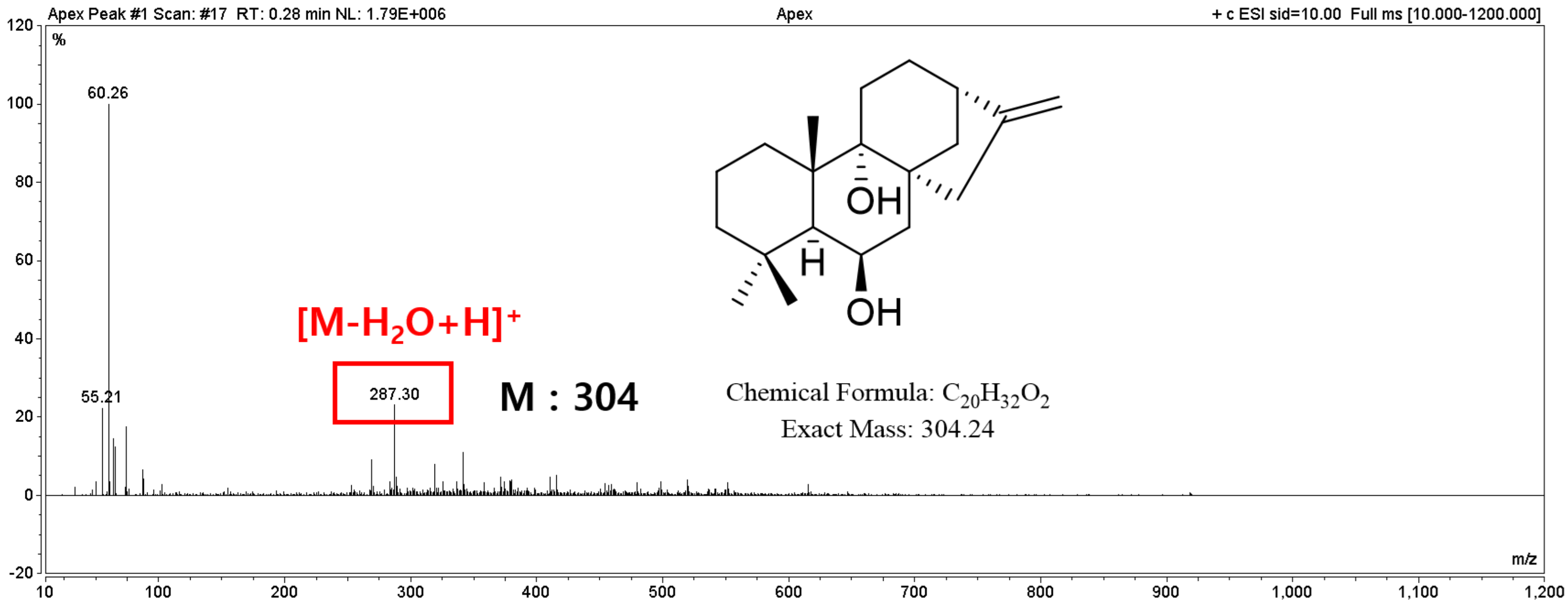


**Figure S36**. LRESIMS data of **5**.

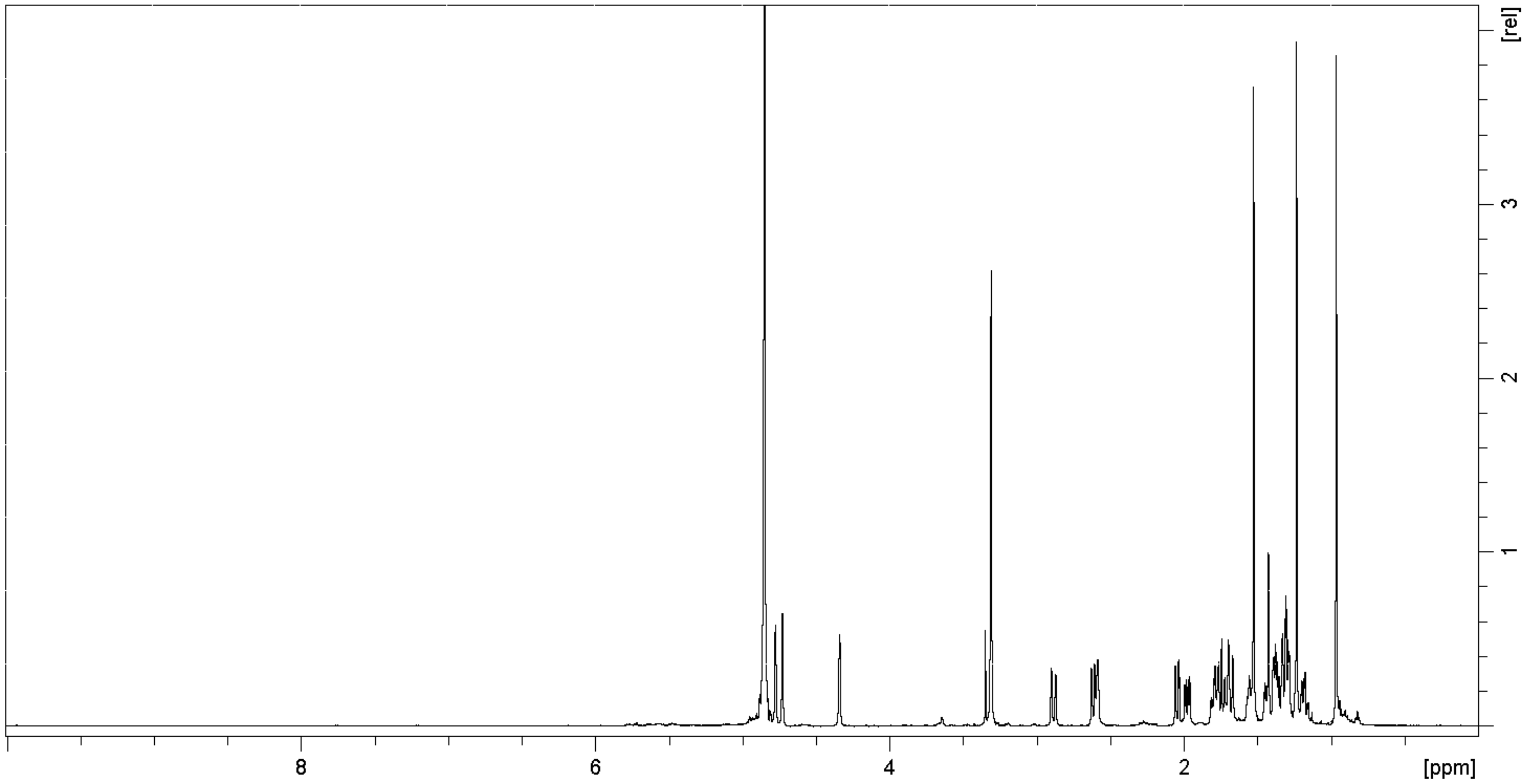


**Figure S37**. $^{1}$H NMR spectrum of **5** ($CD_3OD$).

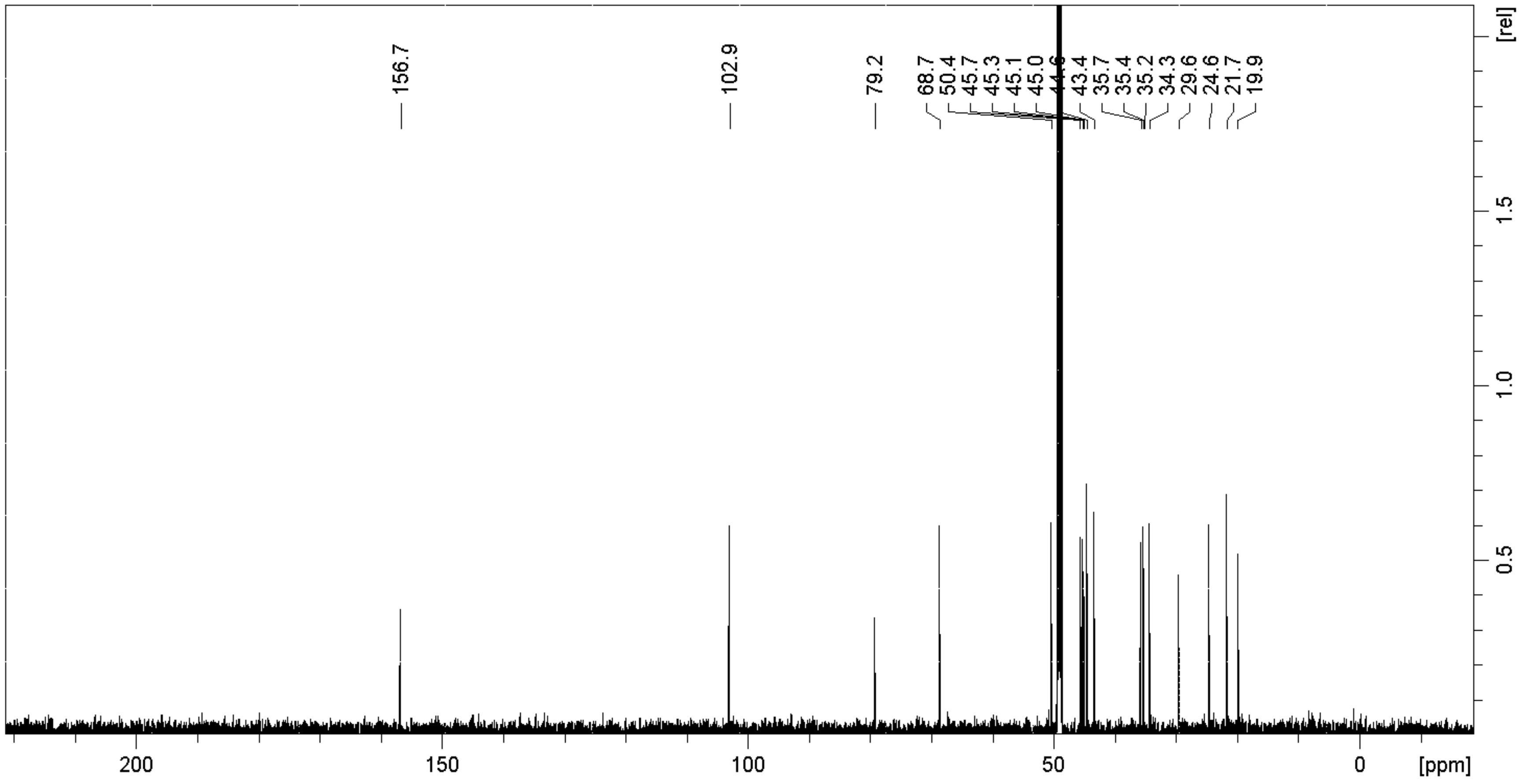


**Figure S38**. $^{13}C$ NMR spectrum of **5** ($CD_3OD$).

| HL-60 | 1 | | 2 | | 3 | | 4 | | 5 | | Doxorubicin | | |
|---|---|---|---|---|---|---|---|---|---|---|---|---|---|
| (μM) | MEAN | SD | MEAN | SD | MEAN | SD | MEAN | SD | MEAN | SD | (μM) | MEAN | SD |
| 30 | 95.8 | 2.6 | 93.6 | 1.8 | 89.3 | 7.0 | 4.9 | 0.1 | 12.4 | 2.7 | 0.1 | 11.0 | 0.7 |
| 10 | 98.1 | 2.2 | 99.4 | 3.9 | 95.6 | 3.2 | 58.6 | 2.0 | 83.5 | 6.1 | 0.03 | 52.7 | 2.9 |
| 3 | 101.5 | 2.3 | 97.7 | 1.5 | 97.6 | 4.6 | 92.0 | 1.2 | 90.3 | 6.2 | 0.01 | 80.5 | 2.8 |
| 1 | 98.7 | 0.6 | 97.5 | 2.9 | 97.9 | 2.6 | 94.2 | 4.6 | 88.6 | 3.5 | 0.003 | 90.7 | 3.3 |
| 0.3 | 96.6 | 2.0 | 99.5 | 3.8 | 101.0 | 3.4 | 94.9 | 1.0 | 87.1 | 2.7 | 0.001 | 86.7 | 3.7 |
| GI50 | **>30** | | **>30** | | **>30** | | **11.210** | | **16.420** | | GI50 | **0.029** | |

| WSU-DLCL2 | 1 | | 2 | | 3 | | 4 | | 5 | | Doxorubicin | | |
|---|---|---|---|---|---|---|---|---|---|---|---|---|---|
| (μM) | MEAN | SD | MEAN | SD | MEAN | SD | MEAN | SD | MEAN | SD | (μM) | MEAN | SD |
| 30 | 93.4 | 2.6 | 95.6 | 3.0 | 101.1 | 3.5 | 0.2 | 0.1 | 0.2 | 0.0 | 0.1 | 4.7 | 0.2 |
| 10 | 101.1 | 2.5 | 101.8 | 2.7 | 101.2 | 2.5 | 2.0 | 0.3 | 15.7 | 3.5 | 0.03 | 9.8 | 1.2 |
| 3 | 101.7 | 5.9 | 100.7 | 2.0 | 98.9 | 4.2 | 61.7 | 4.0 | 90.3 | 4.6 | 0.01 | 43.4 | 4.8 |
| 1 | 103.5 | 3.6 | 102.7 | 2.6 | 96.7 | 1.5 | 88.0 | 1.8 | 93.5 | 4.8 | 0.003 | 81.3 | 1.8 |
| 0.3 | 97.6 | 3.4 | 104.6 | 1.3 | 96.6 | 3.1 | 90.7 | 1.1 | 83.2 | 7.6 | 0.001 | 85.1 | 6.2 |
| GI50 | **>30** | | **>30** | | **>30** | | **3.477** | | **5.928** | | GI50 | **0.008** | |

| K562 | 1 | | 2 | | 3 | | 4 | | 5 | | Doxorubicin | | |
|---|---|---|---|---|---|---|---|---|---|---|---|---|---|
| (μM) | MEAN | SD | MEAN | SD | MEAN | SD | MEAN | SD | MEAN | SD | (μM) | MEAN | SD |
| 30 | 86.2 | 1.6 | 53.4 | 2.2 | 87.7 | 2.6 | 38.7 | 1.4 | 14.7 | 0.9 | 1.0 | 6.6 | 0.3 |
| 10 | 96.9 | 1.8 | 84.3 | 2.8 | 95.9 | 1.5 | 67.7 | 2.5 | 57.6 | 2.0 | 0.3 | 27.3 | 2.9 |
| 3 | 109.8 | 5.2 | 98.4 | 3.9 | 99.7 | 4.8 | 87.9 | 3.6 | 96.1 | 3.5 | 0.1 | 54.7 | 1.6 |
| 1 | 113.3 | 3.4 | 109.7 | 3.0 | 102.2 | 3.1 | 98.3 | 0.9 | 99.4 | 2.1 | 0.03 | 68.8 | 1.4 |
| 0.3 | 114.0 | 2.6 | 114.3 | 1.1 | 104.1 | 1.5 | 96.5 | 7.0 | 98.2 | 1.2 | 0.01 | 84.6 | 1.0 |
| GI50 | **>30** | | **>30** | | **>30** | | **19.640** | | **11.900** | | GI50 | **0.094** | |

| U266 | 1 | | 2 | | 3 | | 4 | | 5 | | Doxorubicin | | |
|---|---|---|---|---|---|---|---|---|---|---|---|---|---|
| (μM) | MEAN | SD | MEAN | SD | MEAN | SD | MEAN | SD | MEAN | SD | (μM) | MEAN | SD |
| 30 | 85.2 | 1.2 | 95.5 | 4.0 | 94.4 | 9.8 | 80.4 | 1.4 | 32.4 | 8.6 | 0.3 | 11.4 | 1.6 |
| 10 | 94.4 | 7.7 | 94.6 | 4.5 | 100.0 | 4.7 | 94.9 | 3.2 | 89.9 | 3.4 | 0.1 | 57.8 | 4.6 |
| 3 | 98.4 | 4.3 | 97.5 | 2.5 | 97.1 | 7.4 | 97.3 | 3.1 | 88.1 | 1.2 | 0.03 | 90.0 | 1.3 |
| 1 | 99.3 | 4.0 | 101.3 | 2.1 | 99.2 | 2.0 | 95.7 | 4.0 | 91.0 | 4.3 | 0.01 | 90.3 | 1.4 |
| 0.3 | 93.5 | 3.1 | 98.5 | 3.9 | 94.2 | 4.8 | 94.9 | 0.6 | 87.2 | 3.2 | 0.003 | 88.1 | 3.5 |
| GI50 | **>30** | | **>30** | | **>30** | | **>30** | | **22.500** | | GI50 | **0.112** | |

| Raji | 1 | | 2 | | 3 | | 4 | | 5 | | Doxorubicin | | |
|---|---|---|---|---|---|---|---|---|---|---|---|---|---|
| (μM) | MEAN | SD | MEAN | SD | MEAN | SD | MEAN | SD | MEAN | SD | (μM) | MEAN | SD |
| 30 | 111.9 | 2.5 | 91.1 | 2.9 | 98.6 | 3.6 | 0.5 | 0.1 | 0.1 | 0.0 | 0.1 | 2.9 | 0.4 |
| 10 | 109.6 | 2.5 | 97.6 | 5.3 | 100.8 | 1.9 | 4.5 | 0.4 | 1.7 | 2.1 | 0.03 | 34.5 | 0.2 |
| 3 | 106.7 | 4.2 | 100.0 | 1.9 | 96.8 | 2.5 | 51.0 | 3.8 | 89.5 | 2.6 | 0.01 | 56.4 | 4.3 |
| 1 | 101.1 | 2.8 | 101.6 | 1.4 | 94.9 | 2.5 | 81.6 | 4.7 | 93.4 | 4.6 | 0.003 | 89.6 | 1.2 |
| 0.3 | 99.8 | 4.6 | 100.3 | 2.4 | 97.4 | 1.4 | 87.8 | 6.9 | 83.1 | 9.0 | 0.001 | 86.5 | 9.3 |
| GI50 | **>30** | | **>30** | | **>30** | | **2.729** | | **4.580** | | GI50 | **0.014** | |

| NALM6 C. G5 | 1 | | 2 | | 3 | | 4 | | 5 | | Doxorubicin | | |
|---|---|---|---|---|---|---|---|---|---|---|---|---|---|
| (μM) | MEAN | SD | MEAN | SD | MEAN | SD | MEAN | SD | MEAN | SD | (μM) | MEAN | SD |
| 30 | 89.4 | 0.6 | 88.2 | 2.9 | 82.4 | 6.2 | 0.5 | 0.1 | 0.1 | 0.0 | 0.1 | 0.1 | 0.0 |
| 10 | 94.6 | 3.6 | 97.5 | 1.1 | 95.6 | 7.3 | 1.7 | 0.3 | 38.4 | 3.5 | 0.03 | 0.0 | 0.1 |
| 3 | 98.4 | 3.7 | 98.4 | 1.5 | 94.6 | 4.4 | 47.6 | 3.4 | 84.1 | 3.1 | 0.01 | 34.5 | 2.9 |
| 1 | 98.8 | 2.9 | 99.1 | 4.3 | 97.9 | 4.8 | 81.9 | 1.7 | 91.7 | 7.8 | 0.003 | 82.7 | 3.6 |
| 0.3 | 93.0 | 1.8 | 96.5 | 1.1 | 97.8 | 3.3 | 91.3 | 1.3 | 90.7 | 4.2 | 0.001 | 86.5 | 1.5 |
| GI50 | **>30** | | **>30** | | **>30** | | **2.591** | | **7.341** | | GI50 | **0.007** | |

| RPMI-8402 | 1 | | 2 | | 3 | | 4 | | 5 | | Doxorubicin | | |
|---|---|---|---|---|---|---|---|---|---|---|---|---|---|
| (μM) | MEAN | SD | MEAN | SD | MEAN | SD | MEAN | SD | MEAN | SD | (μM) | MEAN | SD |
| 30 | 83.2 | 2.5 | 88.1 | 1.3 | 92.4 | 2.9 | 4.3 | 0.9 | 0.2 | 0.0 | 0.3 | 2.3 | 0.4 |
| 10 | 92.2 | 3.4 | 88.9 | 2.3 | 100.0 | 1.3 | 43.2 | 3.9 | 62.4 | 1.7 | 0.1 | 11.6 | 1.5 |
| 3 | 93.2 | 4.0 | 92.8 | 1.4 | 100.0 | 2.4 | 89.1 | 3.7 | 97.3 | 4.8 | 0.03 | 65.0 | 2.3 |
| 1 | 90.8 | 1.8 | 95.5 | 3.6 | 97.3 | 5.3 | 91.8 | 3.7 | 97.3 | 0.8 | 0.01 | 77.6 | 1.3 |
| 0.3 | 88.2 | 3.1 | 90.2 | 2.5 | 98.2 | 4.2 | 94.8 | 3.5 | 91.9 | 3.3 | 0.003 | 86.5 | 1.6 |
| GI50 | **>30** | | **>30** | | **>30** | | **8.507** | | **11.090** | | GI50 | **0.035** | |

| RPMI-1788 | 1 | | 2 | | 3 | | 4 | | 5 | | Doxorubicinl | | |
|---|---|---|---|---|---|---|---|---|---|---|---|---|---|
| (μM) | MEAN | SD | MEAN | SD | MEAN | SD | MEAN | SD | MEAN | SD | (μM) | MEAN | SD |
| 30 | 88.4 | 3.4 | 93.3 | 3.0 | 96.1 | 6.5 | 13.0 | 1.9 | 0.2 | 0.1 | 0.1 | 9.2 | 0.1 |
| 10 | 95.8 | 3.4 | 99.8 | 2.2 | 105.9 | 4.3 | 23.6 | 3.6 | 44.6 | 4.3 | 0.03 | 25.2 | 1.1 |
| 3 | 92.5 | 2.7 | 99.2 | 6.3 | 103.3 | 4.9 | 62.0 | 2.6 | 99.3 | 2.8 | 0.01 | 55.4 | 1.1 |
| 1 | 93.6 | 2.6 | 97.9 | 3.7 | 107.5 | 3.3 | 91.0 | 3.6 | 101.9 | 3.1 | 0.003 | 85.8 | 2.5 |
| 0.3 | 101.6 | 4.4 | 94.8 | 6.7 | 108.3 | 7.6 | 99.9 | 3.2 | 97.1 | 3.6 | 0.001 | 85.9 | 7.3 |
| GI50 | **>30** | | **>30** | | **>30** | | **4.519** | | **9.559** | | GI50 | **0.012** | |

| ACHN | 1 | | 2 | | 3 | | 4 | | 5 | | Adriamycin | | |
|---|---|---|---|---|---|---|---|---|---|---|---|---|---|
| (μM) | MEAN | SD | MEAN | SD | MEAN | SD | MEAN | SD | MEAN | SD | (uM) | MEAN | SD |
| 30 | 77.81 | 4.64 | 77.36 | 2.65 | 81.78 | 3.22 | −5.21 | 2.05 | 86.93 | 2.22 | 3 | 2.39 | 9.00 |
| 10 | 82.41 | 5.32 | 86.32 | 3.76 | 85.47 | 6.93 | 19.08 | 2.70 | 88.79 | 1.49 | 1 | 14.84 | 8.09 |
| 3 | 86.78 | 4.68 | 88.70 | 2.80 | 93.63 | 2.90 | 70.33 | 8.65 | 91.29 | 2.65 | 0.3 | 35.43 | 6.13 |
| 1 | 94.14 | 3.73 | 91.90 | 6.44 | 95.33 | 3.40 | 91.92 | 7.27 | 94.39 | 4.43 | 0.1 | 78.68 | 2.23 |
| 0.3 | 97.28 | 3.38 | 96.84 | 1.75 | 97.16 | 2.47 | 97.06 | 3.28 | 98.35 | 1.51 | 0.03 | 95.63 | 3.11 |
| GI50 | >30 | | >30 | | >30 | | 4.578 | | >30 | | GI50 | 0.222 | |

| MDA-MB-231 | 1 | | 2 | | 3 | | 4 | | 5 | | Adriamycin | | |
|---|---|---|---|---|---|---|---|---|---|---|---|---|---|
| (μM) | MEAN | SD | MEAN | SD | MEAN | SD | MEAN | SD | MEAN | SD | (uM) | MEAN | SD |
| 30 | 79.05 | 8.55 | 80.38 | 9.05 | 79.97 | 8.46 | −7.82 | 6.06 | 75.75 | 1.39 | 3 | 4.47 | 5.03 |
| 10 | 85.56 | 4.30 | 83.66 | 3.64 | 83.93 | 8.03 | 28.42 | 4.16 | 80.24 | 6.91 | 1 | 15.32 | 6.81 |
| 3 | 89.50 | 4.88 | 86.55 | 3.37 | 88.81 | 5.21 | 77.17 | 2.54 | 91.62 | 4.38 | 0.3 | 31.05 | 4.75 |
| 1 | 92.81 | 6.71 | 97.76 | 7.36 | 94.61 | 5.72 | 94.78 | 5.09 | 93.70 | 2.19 | 0.1 | 79.44 | 5.82 |
| 0.3 | 100.65 | 4.37 | 98.58 | 3.17 | 99.43 | 2.93 | 96.03 | 3.67 | 98.14 | 2.88 | 0.03 | 96.86 | 4.03 |
| GI50 | >30 | | >30 | | >30 | | 5.730 | | >30 | | GI50 | 0.210 | |

| PC-3 | 1 | | 2 | | 3 | | 4 | | 5 | | Adriamycin | | |
|---|---|---|---|---|---|---|---|---|---|---|---|---|---|
| (μM) | MEAN | SD | MEAN | SD | MEAN | SD | MEAN | SD | MEAN | SD | (uM) | MEAN | SD |
| 30 | 83.17 | 4.85 | 78.34 | 6.13 | 81.42 | 6.71 | −8.88 | 4.49 | 77.26 | 4.99 | 3 | 7.41 | 5.25 |
| 10 | 87.00 | 6.03 | 82.91 | 3.16 | 86.67 | 2.21 | 36.84 | 5.21 | 84.81 | 9.58 | 1 | 29.71 | 5.12 |
| 3 | 88.58 | 5.08 | 84.11 | 3.34 | 87.45 | 8.68 | 79.57 | 8.73 | 91.66 | 7.58 | 0.3 | 42.03 | 5.32 |
| 1 | 94.88 | 5.69 | 86.32 | 5.46 | 92.47 | 2.90 | 87.95 | 5.71 | 97.84 | 4.21 | 0.1 | 89.21 | 3.94 |
| 0.3 | 98.03 | 3.52 | 95.07 | 6.78 | 97.42 | 3.78 | 95.45 | 6.22 | 98.68 | 1.16 | 0.03 | 97.25 | 4.09 |
| GI50 | >30 | | >30 | | >30 | | 6.498 | | >30 | | GI50 | 0.106 | |

| NUGC-3 | 1 | | 2 | | 3 | | 4 | | 5 | | Adriamycin | | |
|---|---|---|---|---|---|---|---|---|---|---|---|---|---|
| (μM) | Mean | SD | Mean | SD | Mean | SD | Mean | SD | Mean | SD | (uM) | **Mean** | **SD** |
| 30 | 79.90 | 2.69 | 85.33 | 6.27 | 83.88 | 5.80 | −12.57 | 1.07 | 78.74 | 3.85 | 3 | 3.74 | 4.77 |
| 10 | 86.62 | 2.52 | 88.90 | 3.46 | 86.68 | 7.50 | 16.19 | 3.87 | 81.14 | 6.03 | 1 | 17.85 | 4.77 |
| 3 | 91.07 | 1.63 | 90.40 | 2.29 | 89.16 | 2.50 | 69.91 | 3.67 | 88.53 | 2.52 | 0.3 | 37.27 | 5.70 |
| 1 | 93.67 | 2.40 | 91.98 | 2.58 | 94.31 | 3.76 | 90.09 | 2.98 | 92.99 | 4.89 | 0.1 | 82.57 | 5.10 |
| 0.3 | 98.55 | 2.12 | 98.60 | 1.47 | 100.07 | 1.46 | 96.98 | 0.95 | 97.83 | 5.57 | 0.03 | 94.67 | 4.03 |
| GI50 | >30 | | >30 | | >30 | | 4.344 | | >30 | | GI50 | 0.245 | |

| NCI-H23 | 1 | | 2 | | 3 | | 4 | | 5 | | Adriamycin | | |
|---|---|---|---|---|---|---|---|---|---|---|---|---|---|
| (μM) | MEAN | SD | MEAN | SD | MEAN | SD | MEAN | SD | MEAN | SD | (uM) | MEAN | SD |
| 30 | 79.44 | 4.51 | 81.24 | 8.20 | 79.05 | 7.73 | −7.06 | 4.78 | 81.88 | 4.31 | 3 | 5.49 | 2.82 |
| 10 | 82.03 | 8.93 | 84.49 | 8.60 | 85.49 | 3.99 | 24.57 | 3.35 | 85.17 | 8.06 | 1 | 21.89 | 4.12 |
| 3 | 86.88 | 3.64 | 88.30 | 7.57 | 90.60 | 4.82 | 74.09 | 8.99 | 90.69 | 5.90 | 0.3 | 28.65 | 4.13 |
| 1 | 92.54 | 3.00 | 94.11 | 4.91 | 95.44 | 4.05 | 90.39 | 2.95 | 97.00 | 3.91 | 0.1 | 83.39 | 8.58 |
| 0.3 | 95.47 | 2.89 | 96.05 | 5.37 | 97.69 | 3.58 | 98.24 | 2.87 | 99.02 | 2.75 | 0.03 | 96.35 | 7.91 |
| GI50 | >30 | | >30 | | >30 | | 5.130 | | >30 | | GI50 | 0.221 | |

| HCT-15 | 1 | | 2 | | 3 | | 4 | | 5 | | Adriamycin | | |
|---|---|---|---|---|---|---|---|---|---|---|---|---|---|
| (μM) | MEAN | SD | MEAN | SD | MEAN | SD | MEAN | SD | MEAN | SD | (uM) | MEAN | SD |
| 30 | 82.29 | 4.61 | 81.29 | 5.99 | 75.60 | 4.89 | −4.85 | 3.10 | 81.92 | 1.12 | 3 | 9.20 | 2.77 |
| 10 | 84.38 | 2.77 | 85.89 | 1.89 | 80.65 | 3.17 | 25.13 | 2.89 | 87.40 | 6.05 | 1 | 18.63 | 3.60 |
| 3 | 90.41 | 3.39 | 86.19 | 2.65 | 88.31 | 1.75 | 81.34 | 4.13 | 89.39 | 2.99 | 0.3 | 45.18 | 3.63 |
| 1 | 94.92 | 2.22 | 96.43 | 4.43 | 91.52 | 4.11 | 86.33 | 2.01 | 94.21 | 2.05 | 0.1 | 87.84 | 3.02 |
| 0.3 | 97.19 | 4.04 | 98.86 | 2.26 | 95.77 | 2.92 | 93.63 | 2.74 | 97.28 | 1.43 | 0.03 | 96.62 | 3.17 |
| GI50 | >30 | | >30 | | >30 | | 5.733 | | >30 | | GI50 | 0.305 | |

| MRC-9 | 1 | | 2 | | 3 | | 4 | | 5 | | Adriamycin | | |
|---|---|---|---|---|---|---|---|---|---|---|---|---|---|
| (μM) | MEAN | SD | MEAN | SD | MEAN | SD | MEAN | SD | MEAN | SD | (uM) | MEAN | SD |
| 30 | 80.95 | 3.63 | 76.06 | 4.24 | 83.45 | 4.18 | 1.29 | 2.77 | 77.49 | 4.89 | 3 | 9.44 | 5.16 |
| 10 | 82.50 | 3.74 | 88.18 | 1.69 | 86.37 | 6.27 | 32.67 | 4.73 | 86.22 | 6.45 | 1 | 27.01 | 1.91 |
| 3 | 90.81 | 2.99 | 91.89 | 1.40 | 91.19 | 4.30 | 88.68 | 6.79 | 91.40 | 2.86 | 0.3 | 40.92 | 7.51 |
| 1 | 92.26 | 3.29 | 93.39 | 5.02 | 94.96 | 4.50 | 93.48 | 4.10 | 94.08 | 2.49 | 0.1 | 86.72 | 4.40 |
| 0.3 | 98.80 | 2.40 | 97.32 | 5.37 | 96.52 | 3.25 | 97.42 | 3.08 | 97.08 | 2.43 | 0.03 | 96.09 | 2.13 |
| GI50 | >30 | | >30 | | >30 | | 7.232 | | >30 | | GI50 | 0.314 | |

**Table S1**. Results of the cytotoxicity test of **1-5**.
